# SpaCut: Transcript‐Aware Morphology Fusion for Robust Cell Segmentation in Subcellular Spatial Transcriptomics

**DOI:** 10.1002/advs.78423

**Published:** 2026-10-29

**Authors:** Yongheng Li, Fangfang Zhu, Wenwen Min

**Affiliations:** ^1^ School of Information Science and Engineering Yunnan University Kunming Yunnan China; ^2^ School of Health and Nursing Yunnan Open University Kunming Yunnan China

**Keywords:** cell segmentation, multimodal deep learning, spatial transcriptomics

## Abstract

Subcellular Spatial Transcriptomics (SST) offers unprecedented opportunities to decipher tissue architecture at subcellular resolution. Consequently, accurate cell segmentation is a fundamental prerequisite for analyzing SST data, as precise transcript assignment is essential for downstream single‐cell interpretation. Existing methods often overlook the spatial organization of transcripts, rely on simplified geometric priors, or struggle to coordinate transcript‐derived features with morphology‐derived spatial guidance. This limitation restricts their adaptability to irregular cell shapes, often leading to transcript misassignment. To address this, we propose SpaCut, a unified deep learning framework designed to effectively integrate transcriptomic feature learning with dynamic morphological guidance. SpaCut comprises a Gene‐Wise Attention (GWA) module and a Nuclei‐Guided Attentive Fusion (NGAF) module. GWA encodes the high‐dimensional transcript map and aggregates features across multiple depths. NGAF uses the encoded transcript features to modulate distance‐based nuclear softmasks and refine nucleus‐anchored cell boundaries through progressive training. Extensive evaluations across seven datasets from diverse platforms (including Xenium, CosMx, MERSCOPE, and Stereo‐seq) demonstrate that SpaCut significantly outperforms state‐of‐the‐art methods in both segmentation accuracy and consistency with single‐cell reference data. The source code is available at https://github.com/wenwenmin/SpaCut.

## Graphical abstract

Subcellular spatial transcriptomics enables high‐resolution mapping of gene expression within tissues, but accurate cell segmentation is essential for assigning transcripts to individual cells. We present SpaCut, a deep learning framework that integrates transcriptomic features with nucleus‐guided morphological information to precisely delineate cell boundaries. Across diverse spatial transcriptomics datasets, SpaCut improves segmentation accuracy and transcript assignment, enabling more reliable single‐cell analysis of tissue architecture.

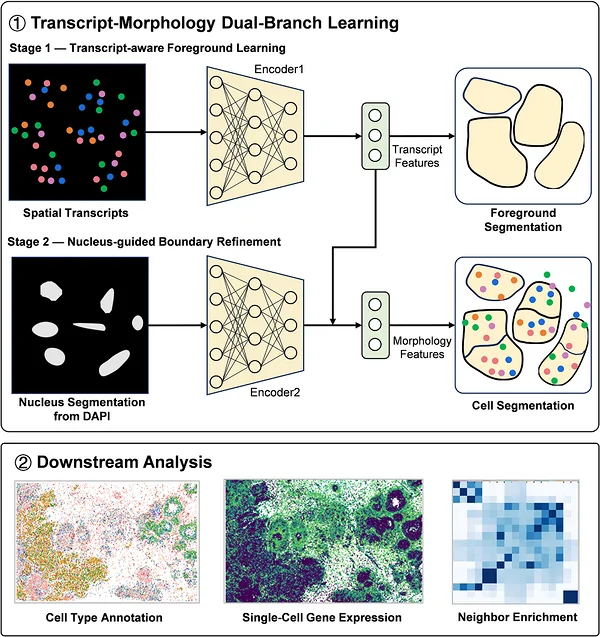

## 1 Introduction

Elucidating how cells organize into tissues and interact within their environment is fundamental to biomedical research [1, 2, 3]. Subcellular Spatial Transcriptomics (SST) technologies enable the measurement of gene expression within intact tissues at subcellular resolution [4, 5]. However, deriving biological insights from these data requires accurately attributing transcripts to individual cells through the segmentation of the spatial domain. The precision of this segmentation directly impacts downstream analyzes, such as cell type annotation and cell–cell communication [6, 7, 8]. Specifically, segmentation errors introduce bias: over‐segmentation incorporates extracellular noise or signals from neighboring cells, distorting spatial gene expression patterns; conversely, under‐segmentation results in the loss of valid transcripts, leading to incomplete gene expression profiles. Therefore, accurate cell segmentation is essential for the effective analysis of SST data [9, 10].

Currently, SST technologies vary significantly in key parameters such as resolution and gene profiling capacity [11]. Imaging‐based platforms, such as Vizgen's MERSCOPE [12] and 10× Genomics' Xenium [13], offer high resolution (∼100nm) and capture efficiency for targeted gene panels. Notably, NanoString's CosMx [14] expands this capability, detecting thousands of genes while maintaining subcellular precision. Meanwhile, sequencing‐based platforms like Stereo‐seq [15] stand out by offering whole transcriptome analysis, capturing over 10000 genes with a resolution of 0.5 μm. This distinction leads to different data characteristics: imaging methods generally prioritize high capture efficiency for specific gene sets, whereas sequencing methods deliver comprehensive genome‐wide profiles [16, 17]. Consequently, developing a unified cell segmentation method that adapts to these diverse data characteristics and resolutions remains a significant challenge.

Existing cell‐segmentation methods for SST can be broadly grouped into three categories: image‐based, transcript‐based, and integrative image–transcript approaches. Image‐based approaches [18, 19, 20, 21, 22, 23] primarily rely on deep neural networks trained on large‐scale annotated cellular images. Leveraging architectures such as U‐Net [24] or Transformers [25], these frameworks extract visual features like staining intensity and texture, determining cell boundaries based solely on visual information. Although tools like Cellpose [18] and StarDist [19] excel at nuclear detection, they are unable to capture complex gene expression patterns outside the nuclear region. This limitation extends to commercial platforms like 10× Xenium and Vizgen, which rely on similar nuclear‐based strategies. Consequently, they often face challenges such as inaccurately large cells resulting from Xenium's default dilation or transcript loss due to Vizgen's conservative segmentation.

Transcript‐based methods instead infer cellular assignments primarily from molecular distributions. Baysor [26] utilizes Markov Random Field modeling to partition molecules based on compositional consistency, allowing it to function independently of histological images. Conversely, ProSeg [27] adopts a simulation‐based Cellular Potts Model. While it uses nuclear centroids for initialization, it primarily drives boundary expansion by optimizing the probabilistic likelihood of transcript distributions. Although these methods excel in regions with poor staining, inferring boundaries solely from transcript distributions often results in imprecise segmentations that do not correspond well to imaging data.

Integrative image–transcript methods seek to overcome the complementary weaknesses of these two categories by incorporating molecular information into morphology‐guided boundary inference. JSTA [28] jointly optimizes cell segmentation and cell‐type annotation using reference expression profiles, whereas SCS [29] combines imaging and sequencing data by learning the relative position of each transcript spot to its cell center. BIDCell [30] introduces biologically informed self‐supervised losses to relate spatial gene expression to nuclear morphology and reference cell‐type information. UCS [31] adopts a two‐stage nucleus‐anchored framework that combines transcript‐derived foreground features with distance‐based nuclear softmasks. Despite these advances, encoding sparse transcript distributions and adaptively integrating them with nucleus‐derived spatial priors remain challenging.

To address these challenges, we propose SpaCut, a transcript‐aware, nucleus‐guided framework for cell segmentation (Figure 1). The Gene–Wise Attention (GWA) module projects raw gene channels into a learned feature space and recalibrates transcript‐derived features to suppress noise and generate a coarse cellular foreground map. The Nuclei‐Guided Attentive Fusion (NGAF) module then uses these features to modulate distance‐based nuclear softmasks and refine nucleus‐anchored boundaries according to local transcript distributions. A progressive training strategy first learns foreground localization and subsequently freezes the transcript encoder while optimizing instance‐specific boundary refinement. Benchmarking against representative methods across seven datasets spanning Xenium, CosMx, MERSCOPE, and Stereo‐seq showed that SpaCut achieved higher segmentation accuracy and greater consistency with single‐cell reference profiles.

**FIGURE 1:**
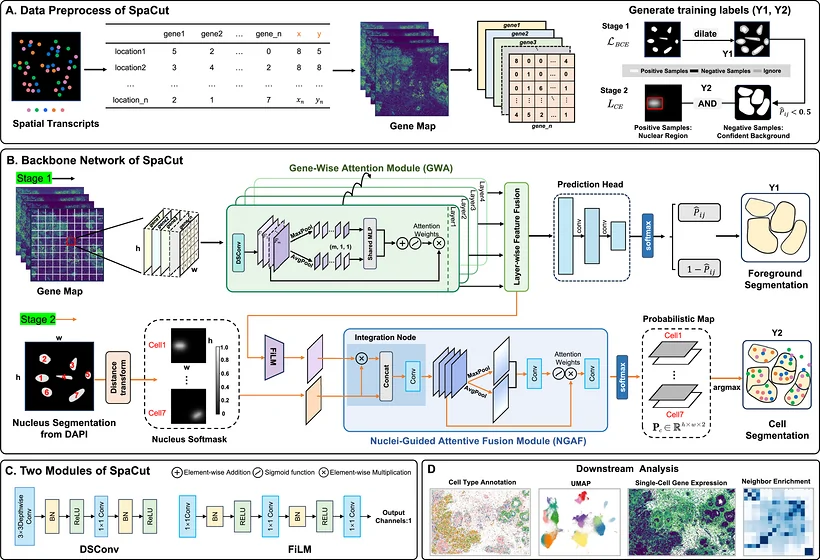
Overview of the SpaCut architecture. (A) Spatial transcriptomics data are transformed into tensor representations, where each gene corresponds to an individual slice (gene map). The input nucleus segmentation is further used to generate training labels ($Y_{1}$ and $Y_{2}$) for Stage 1 and Stage 2, respectively. (B) Backbone network of SpaCut. (C) Two key modules of SpaCut, including the depthwise separable convolution (DSConv) module and the feature‐wise linear modulation (FiLM) module. (D) Downstream analysis enabled by SpaCut.

## 2 Results

### 2.1 Overview of SpaCut

To achieve accurate cell segmentation from SST data, we propose SpaCut. Designed to leverage both high‐dimensional gene expression maps and nucleus segmentation from nuclei staining, the network first employs a Gene‐Wise Attention (GWA) Module to encode the gene expression data into refined features. This module functions as a robust encoder, capturing spatial context while adaptively re‐weighting gene importance. The encoded features are then utilized in two sequential stages. First, a classification head generates a coarse foreground localization map, distinguishing cellular areas from the background. Simultaneously, discrete nuclei segmentations are smoothed into continuous softmasks via signed distance mapping. Subsequently, the Nuclei‐Guided Attentive Fusion (NGAF) Module integrates the nucleus softmask as a spatial prior. It employs a FiLM‐inspired mechanism where gene features generate a spatial coefficient map to dynamically modulate this prior, guiding the expansion from the nucleus to the precise cellular boundaries. SpaCut adopts a progressive training strategy: it first uses the Weighted Cross‐Entropy Loss ($L_{\text{fg}}$) to train the foreground localization; it then freezes the encoded features and employs the Weighted Cross‐Entropy Loss ($L_{\text{cell}}$) to train the NGAF module for individual cell boundary refinement. By jointly optimizing for foreground consistency and individual cell precision, SpaCut effectively produces robust cell segmentation.

### 2.2 SpaCut Enables Accurate Cell Segmentation Across Diverse Spatial Transcriptomics Platforms and Tissue Architectures

To comprehensively assess SpaCut's generalization capability, we evaluated its performance across seven datasets spanning four leading SST platforms: 10× Genomics Xenium, NanoString CosMx SMI, Vizgen MERSCOPE, and BGI Stereo‐seq. These datasets cover epithelial, stromal, and neural tissues, capturing a broad range of cellular morphologies and tissue architectures. This diversity introduces substantial segmentation challenges, specifically complex cellular morphologies and varying data quality across different technologies. SpaCut effectively handles this biological and technical variability, achieving accurate and consistent cell segmentation across these highly heterogeneous platforms.

The proposed SpaCut framework demonstrates robust and accurate performance in cell segmentation across diverse SST platforms. In Xenium Human Pancreas and Mouse Colon datasets (Figure 2A,B), where 10× Genomics annotations serve as the ground truth, SpaCut produces segmentation masks that closely align with reference boundaries. Compared with UCS and Baysor(prior), it yields smoother and biologically coherent boundaries while effectively avoiding the over‐segmentation observed in UCS. Such robust performance is similarly observed in the Xenium Lung Cancer dataset (Figure 2C), where SpaCut precisely delineates elongated fibroblast‐like cells and irregular stromal cells in the tumor microenvironment, outperforming competing methods that often distort cellular morphology. In the Stereo‐seq Mouse Brain dataset (Figure 2D), SpaCut maintains compact and morphologically consistent segmentations under sparse transcript density and high spatial resolution—conditions where transformer‐based models such as SCS tend to produce fragmented boundaries. In the Vizgen Ovarian Cancer dataset (Figure 2E), we leveraged Vizgen's underestimated default segmentation results as “nuclei” inputs to assess cross‐platform adaptability. By reinterpreting segmentation masks as nuclear priors, SpaCut improves transcript coverage and accurately incorporates regions of high gene expression (P<0.001), thereby increasing the number of assigned transcripts per cell for downstream analyzes. Finally, in the CosMx Human Pancreas dataset (Figure 2F), SpaCut achieves results highly consistent with NanoString's full‐cell reference annotations, despite relying solely on nuclear fluorescence and transcript information. Additional same‐region visual comparisons across multiple datasets and platforms are provided in Figures S1 and S2. These panels show that SpaCut retains coherent local boundaries under diverse microscopy backgrounds and transcript‐density patterns. Collectively, these results highlight SpaCut's strong cross‐modality generalization and robust performance under nuclei‐only staining conditions, which is a crucial capability for most SST datasets lacking whole‐cell stains.

**FIGURE 2:**
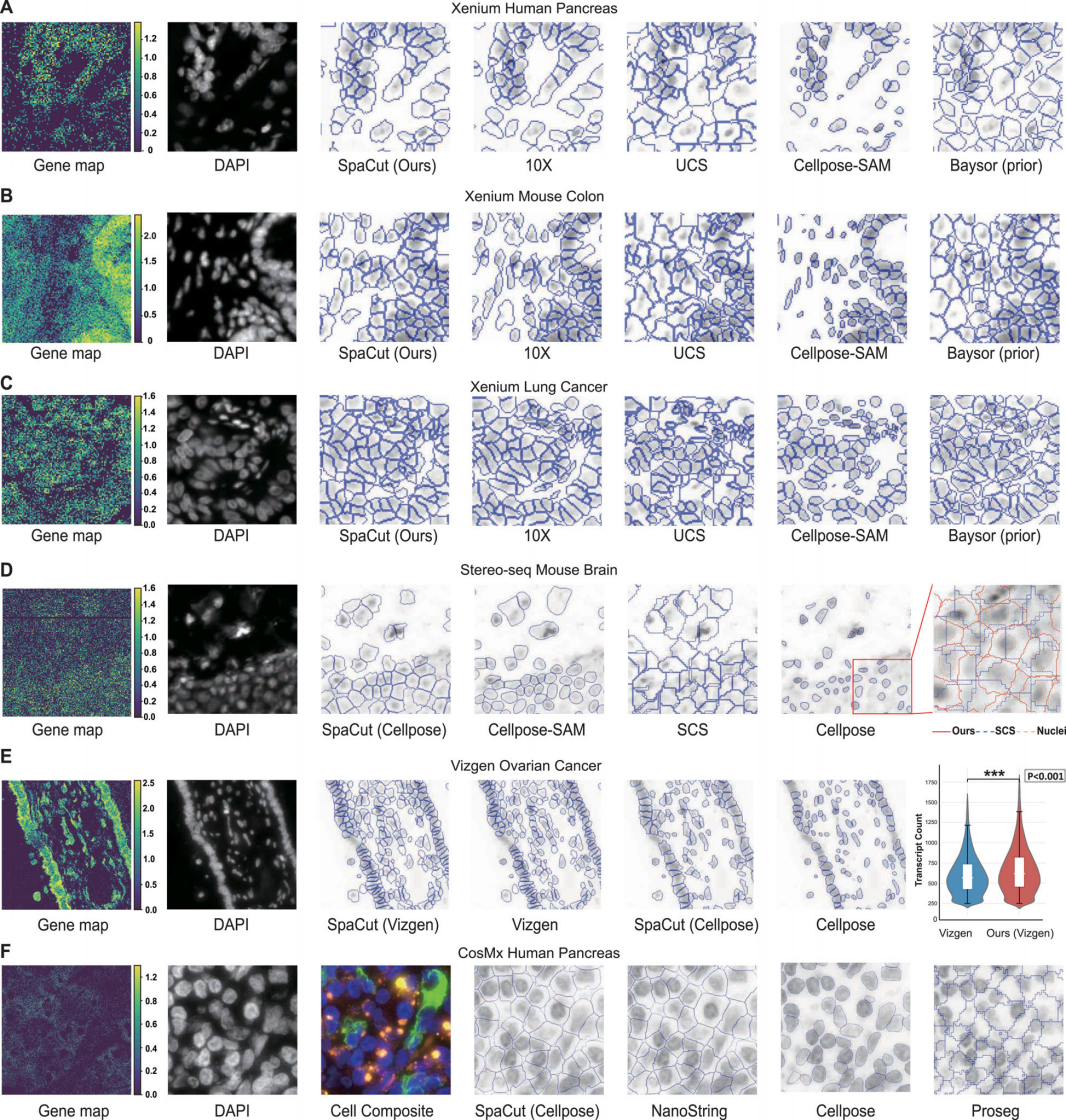
Generalizability of SpaCut across six datasets. (A) Xenium human pancreas dataset; (B) Xenium mouse colon dataset; (C) Xenium human lung cancer dataset; (D) Stereo‐seq mouse brain dataset; (E) Vizgen ovarian cancer dataset; and (F) CosMx human pancreas dataset. SpaCut achieves accurate cell segmentation across diverse spatial transcriptomics platforms, consistently outperforming existing methods. Notations such as “SpaCut (10×)”, “SpaCut (Cellpose)”, and “SpaCut (Vizgen)” indicate that the nuclei segmentation inputs are derived from the corresponding sources.

We further evaluated the robustness of SpaCut on the Xenium Breast Cancer dataset by randomly subsampling transcripts at varying ratios (80%, 60%, 40%, and 20%) (Figure 3A). This approach intentionally introduces signal sparsity to assess algorithmic stability under challenging, low‐quality conditions. Using the original dataset (100% transcripts) as the ground truth, we benchmarked the performance of SpaCut against BIDCell and UCS across these subsampled levels. SpaCut maintained robust performance and remained consistent with the H&E image even at the lowest signal level (20%) (Figure 3B). This stability is corroborated by the F1‐score analysis(Figure 3C), demonstrating its ability to preserve segmentation accuracy under noisy conditions. In contrast, competing methods showed a marked decline as signal density decreased. BIDCell suffered a sharp drop in accuracy with increasingly fragmented boundaries, while UCS exhibited moderate sensitivity to reduced signals. These results highlight SpaCut's robustness to handle noisy and incomplete datasets, maintaining reliable cell segmentation in various spatial transcriptomics datasets.

**FIGURE 3:**
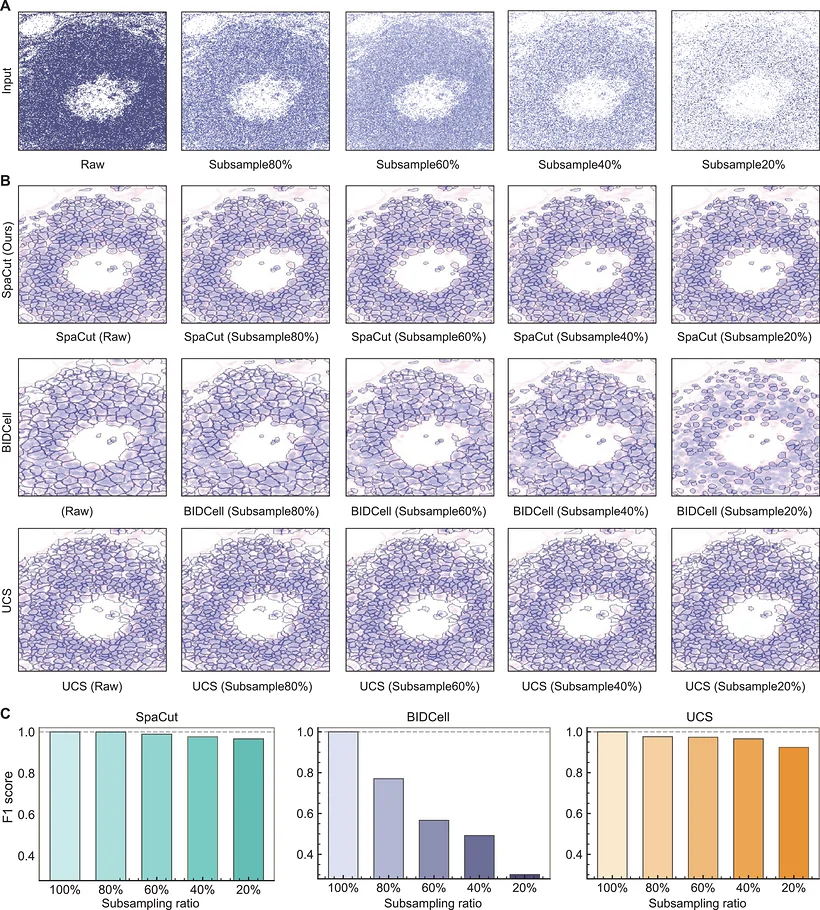
Robustness evaluation under transcript‐level subsampling on the Xenium breast cancer dataset. (A) To simulate varying signal intensities, transcriptomic data were subsampled to 80%, 60%, 40%, and 20% for *ERBB2* visualization. (B) Segmentation results of SpaCut, BIDCell, and UCS under different subsampling ratios. (C) Quantitative F1 score comparison demonstrating that SpaCut maintains consistently high accuracy across all subsampling levels, whereas BIDCell shows a sharp performance drop and UCS exhibits moderate sensitivity to reduced signals.

SpaCut also remained robust to transcript corruption on Xenium Human Lung Cancer, achieving the highest instance F1 and AJI among the three evaluated methods across all tested conditions. Its F1 decreased from 0.8227 on the clean input to 0.7767 after 80% transcript loss and 0.7765 after adding 80% background transcripts (Figures S3 and S4). Together with the cross‐platform benchmarks, these results support the generalizability and robustness of SpaCut across diverse SST datasets.

### 2.3 SpaCut Achieves State‐of‐the‐Art Segmentation Accuracy on Xenium Datasets

To comprehensively evaluate the performance of SpaCut, we conducted rigorous benchmarking on three representative Xenium spatial transcriptomics datasets, including Human Lung Cancer, Human Pancreas (FFPE), and Mouse Colon (FF) datasets. Unlike most publicly available Xenium data, which often lack reliable ground truth annotations, the Xenium 2.0 platform provides dual‐channel fluorescence imaging that simultaneously stains both nuclei and cell boundaries. This dual labeling provides reliable ground truth for segmentation evaluation. Using this reference, we compared SpaCut with a suite of representative segmentation methods. Segmentation performance was quantified using two established metrics including the Aggregated Jaccard Index (AJI) and the F1 score at an Intersection over Union (IoU) threshold of 0.4 (See Table 1).

**TABLE 1:** Quantitative comparison of different segmentation methods on three Xenium datasets. The best and second‐best results are highlighted in **bold** and underlined, respectively.

Datasets	Human lung cancer		Human pancreas		Mouse colon	
methods	AJI	F1 Score	AJI	F1 Score	AJI	F1 Score
Cellpose	0.6314 ±.0015	0.4881 ±.0019	0.6175 ±.0013	0.2809 ±.0015	0.4351 ±.0009	0.2861 ±.0015
Cellpose3	0.6633 ±.0018	0.3399 ±.0017	$\underline{0.6386}$ $\;\pm \;\underline{.0016}$	0.1739 ±.0008	0.4656 ±.0016	0.1818 ±.0009
DeepCell	0.6108 ±.0051	0.4310 ±.0159	0.5937 ±.0047	0.2727 ±.0088	0.4328 ±.0029	0.2522 ±.0181
MEDIAR	0.6577 ±.0059	0.3595 ±.0113	0.6356 ±.0061	0.1885 ±.0052	$\underline{0.4664}$ $\;\pm \;\underline{.0073}$	0.1986 ±.0103
SAM	0.4684 ±.0019	0.3701 ±.0029	0.4328 ±.0972	0.2340 ±.0214	0.3039 ±.0164	0.2088 ±.0009
StarDist	0.6066 ±.0070	0.3097 ±.0134	0.5922 ±.0061	0.1745 ±.0045	0.4149 ±.0072	0.1546 ±.0065
Cellpose‐SAM	0.6236 ±.0041	0.5532 ±.0034	0.5912 ±.0089	0.3809 ±.0100	0.4252 ±.0078	0.2805 ±.0112
UCS	0.4473 ±.0079	0.6913 ±.0044	0.5030 ±.0451	$\underline{0.5286}$ $\;\pm \;\underline{.0243}$	0.4239 ±.0140	$\underline{0.6452}$ $\;\pm \;\underline{.0084}$
Baysor (no prior)	0.3004 ±.0319	0.2818 ±.0325	0.2853 ±.0141	0.1274 ±.0006	0.1884 ±.0029	0.0874 ±.0002
Baysor (with prior)	$\underline{0.6640}$ $\;\pm \;\underline{.0003}$	$\underline{0.7619}$ $\;\pm \;\underline{.0004}$	0.4325 ±.0133	0.2569 ±.0003	0.3334 ±.0022	0.3542 ±.0015
**SpaCut**	0.7461 ±.0059	0.9381 ±.0038	0.7454 ±.0040	0.8953 ±.0057	0.6151 ±.0211	0.8980 ±.0127

Across all evaluation metrics and datasets, SpaCut consistently demonstrated superior performance (Table 1, Figure 4). Specifically, it achieved leading AJI scores of 0.7461, 0.7454, and 0.6151 on the lung, pancreas, and colon datasets, respectively, substantially outperforming all competitors and reflecting higher agreement with the reference annotations. The corresponding F1 scores—0.9381, 0.8953, and 0.8980—further confirm its advantage. As shown in Figure 4, SpaCut consistently achieves higher F1 scores across a wide range of IoU thresholds. This stable advantage under different matching thresholds demonstrates the robustness of our model in accurately identifying cell boundaries. In contrast, image‐based approaches showed lower performance across these datasets. These methods rely primarily on nuclear morphology, whereas SpaCut additionally integrates spatial transcriptomic information to support cell‐boundary delineation.

**FIGURE 4:**
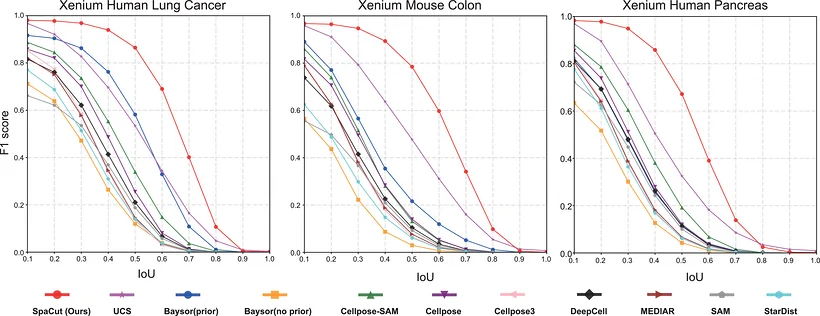
Instance‐level F1–IoU curves on Xenium Human Lung Cancer, Xenium Human Pancreas, and Xenium Mouse Colon. The horizontal axis denotes the IoU matching threshold, and the vertical axis shows the corresponding instance‐level F1 score. At each threshold, predicted and reference cell instances are paired using maximum‐cardinality one‐to‐one matching, with unmatched predictions and references counted as false positives and false negatives, respectively. Increasing the IoU threshold imposes a stricter overlap criterion; methods that retain higher F1 scores therefore achieve more accurate cell‐instance detection and boundary delineation.

Qualitative results further support these quantitative observations. As shown in Figure 2A–C, SpaCut generates sharp and continuous cell boundaries across various tissue types. In regions with dense cellular packing, it accurately preserves cell morphology, while existing methods often yield fragmented or blurry boundaries. This improvement stems from SpaCut's joint modeling of local morphological features and spatial transcriptomic signals, which enables precise cell segmentation even in complex and heterogeneous tissue environments.

In summary, both quantitative and qualitative evaluations demonstrate that SpaCut achieves state‐of‐the‐art segmentation performance on Xenium datasets. Its effectiveness arises from the joint modeling of morphological and transcriptomic spatial features, leading to accurate cell segmentation. This capability provides a strong foundation for reliable downstream spatial transcriptomics analyzes.

### 2.4 SpaCut Achieves Multi‐Scale Morphological and Transcriptomic Consistency on Xenium Breast Cancer

To comprehensively evaluate the performance of SpaCut in cell segmentation and annotation, we conducted a multi‐scale benchmarking analysis using the Xenium Human Breast Cancer dataset (Figure 5). As shown in Figure 5A, SpaCut shows stronger alignment with the H&E staining compared to existing methods. Regions with clear nuclear staining are accurately segmented, while low‐contrast or background areas are correctly ignored—errors that are frequently observed in the segmentation results of 10×. Baysor (prior) tends to generate overly smooth and enlarged masks that merge adjacent cells, while Baysor (no prior) often produces fragmented boundaries. Cellpose‐SAM and Cellpose3 achieve nucleus‐centered segmentation that aligns well with dark‐stained regions and effectively separates close nuclei, but they frequently undersegment cytoplasmic or lightly stained areas.

**FIGURE 5:**
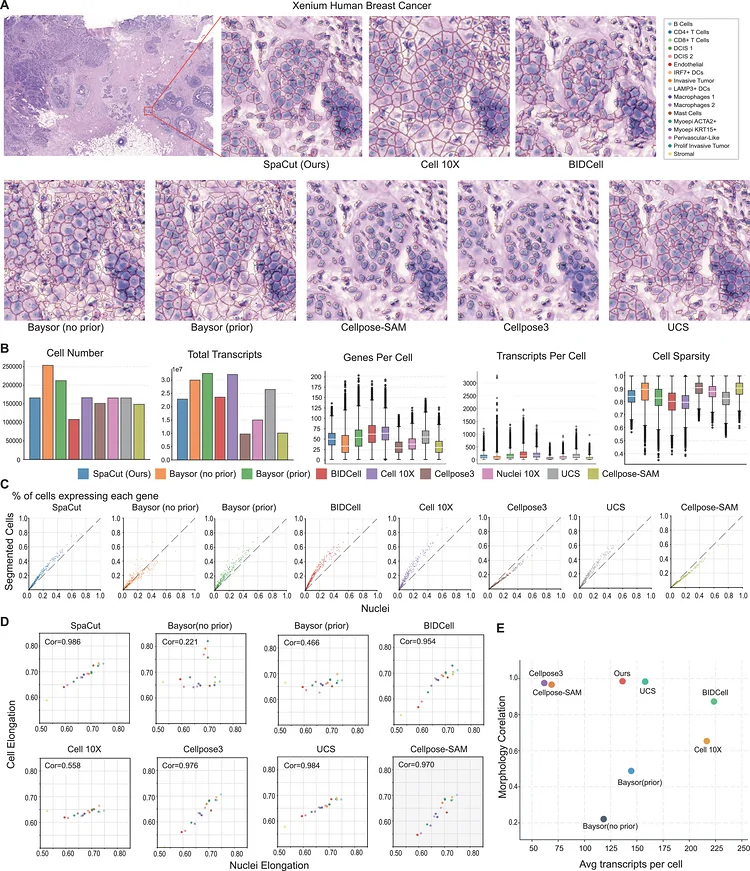
SpaCut demonstrates accurate transcriptional and morphological consistency on the Xenium breast cancer dataset. (A) Visual comparison of segmentation boundaries overlaid on H&E images. SpaCut exhibits better morphological consistency with histological structures compared to baselines. (B) Quantitative statistics comparing cell count, transcript recovery, genes/transcripts per cell, and sparsity across methods. (C) Gene‐level quality assessment. Scatter plots display the fraction of cells expressing each gene in segmented cells (y‐axis) vs. nuclei (x‐axis). (D) Morphological validation. Left: Correlation between cell and nucleus elongation across cell types. (E) Overall morphology correlation plotted against average transcripts per cell, demonstrating SpaCut's effective balance between transcriptional sensitivity and morphological preservation.

To quantitatively evaluate segmentation performance, we further analyzed both cell‐level and gene‐level statistics (Figure 5B,C). At the cell level, five representative metrics—cell count, total transcripts, genes per cell, transcripts per cell, and cell sparsity were used to examine the trade‐off between segmentation outcomes and transcripts capture. Baysor (no prior) tends to over‐segment, producing numerous small cells with sparse transcript counts, while the nuclei dilation method used in the 10× official pipeline generate segment large cells that include background or neighboring signals. In contrast, SpaCut achieves a balanced segmentation profile, recovering a biologically consistent number of cells with stable per‐cell gene and transcript distributions. At the gene level, we compared the proportion of cells expressing each gene between segmented masks and nuclei‐based expression to assess cytoplasmic transcript recovery. Methods with points near the diagonal recover little beyond nuclei, whereas SpaCut shifts more genes above the diagonal, indicating improved cytoplasmic transcript recovery and transcriptome completeness. Across the three Xenium datasets, SpaCut achieved the highest correct cytoplasmic recovery among non‐reference methods (0.6964–0.7324) and the highest assignment F1 (0.7619–0.8371). Although several conservative image‐only methods captured fewer extracellular transcripts, they recovered substantially fewer correct cytoplasmic transcripts. These results support a favorable recovery–contamination balance rather than an advantage driven solely by larger masks (Table S1 and Figure S5).

Finally, we examined how well each method preserves cellular morphology by correlating nuclear elongation (x‐axis) with cell‐mask elongation (y‐axis) (Figure 5D). Image‐based models such as Cellpose and Cellpose‐SAM show high morphology correlations but exhibit lower average transcript counts per cell, suggesting that their segmentations primarily follow nuclear boundaries and underrepresent cytoplasmic regions. In contrast, SpaCut shows strong nuclear–cell shape agreement while achieving comparable transcripts per cell than competing methods(Figure 5E), suggesting improved transcript recovery beyond the nucleus. UCS and BIDCell also display consistent nuclear–cell coupling, whereas Baysor (no prior) exhibits weak correlation and Cell 10X achieves only moderate agreement. Before post‐processing, shuffling or removing transcripts reduced instance F1 from 0.8638 to 0.7389 and 0.7032, respectively, and the full model outperformed fixed‐dilation and analytic‐softmask controls. SpaCut also achieved the largest mean difference between boundary‐expression similarity to the cell's own nucleus and that to adjacent nuclei, although its advantage over fixed dilation was modest. Together, these results support transcript‐dependent boundary refinement beyond simple geometric expansion (Table S2 and Figures S6 and S7). Collectively, these analyzes confirm that SpaCut preserves accurate cell morphology while achieving improved transcript capture, producing coherent cell boundaries that reflect both cellular structure and gene expression.

### 2.5 SpaCut Improves Spatial Cell‐type Annotation with scRNA‐seq Data

An accurate cell segmentation method should generate cellular gene expression profiles closely matching the single‐cell reference dataset. To this end, we integrated SpaCut‐segmented cells with tissue‐matched scRNA‐seq data using scvi‐tools [32]. The joint UMAP embedding (Figure 6A) reveals superior cross‐modality alignment between SpaCut‐segmented cells and the single‐cell reference. As illustrated in Figure 6B, SpaCut enables the precise identification of distinct cancer regions. Specifically, it accurately distinguishes the Invasive Tumor (Orange) from the DCIS subtypes (Green and Pink). This is consistent with the manually annotated cancer regions shown in (Figure 6B). We further quantitatively validated this performance through transcriptomic consistency analysis. Specifically, we calculated the Pearson correlation coefficient (PCC) between the average gene expression profiles of each cell type derived from the segmentation results and their corresponding clusters in the scRNA‐seq reference. This comparison reveals that SpaCut achieves higher correlations than baseline methods (Figure 6C). This metric serves as a quantitative benchmark to assess the consistency between the segmented spatial expression patterns and the single‐cell reference. Cell‐type proportion concordance is additionally summarized in Figure 6D.

**FIGURE 6:**
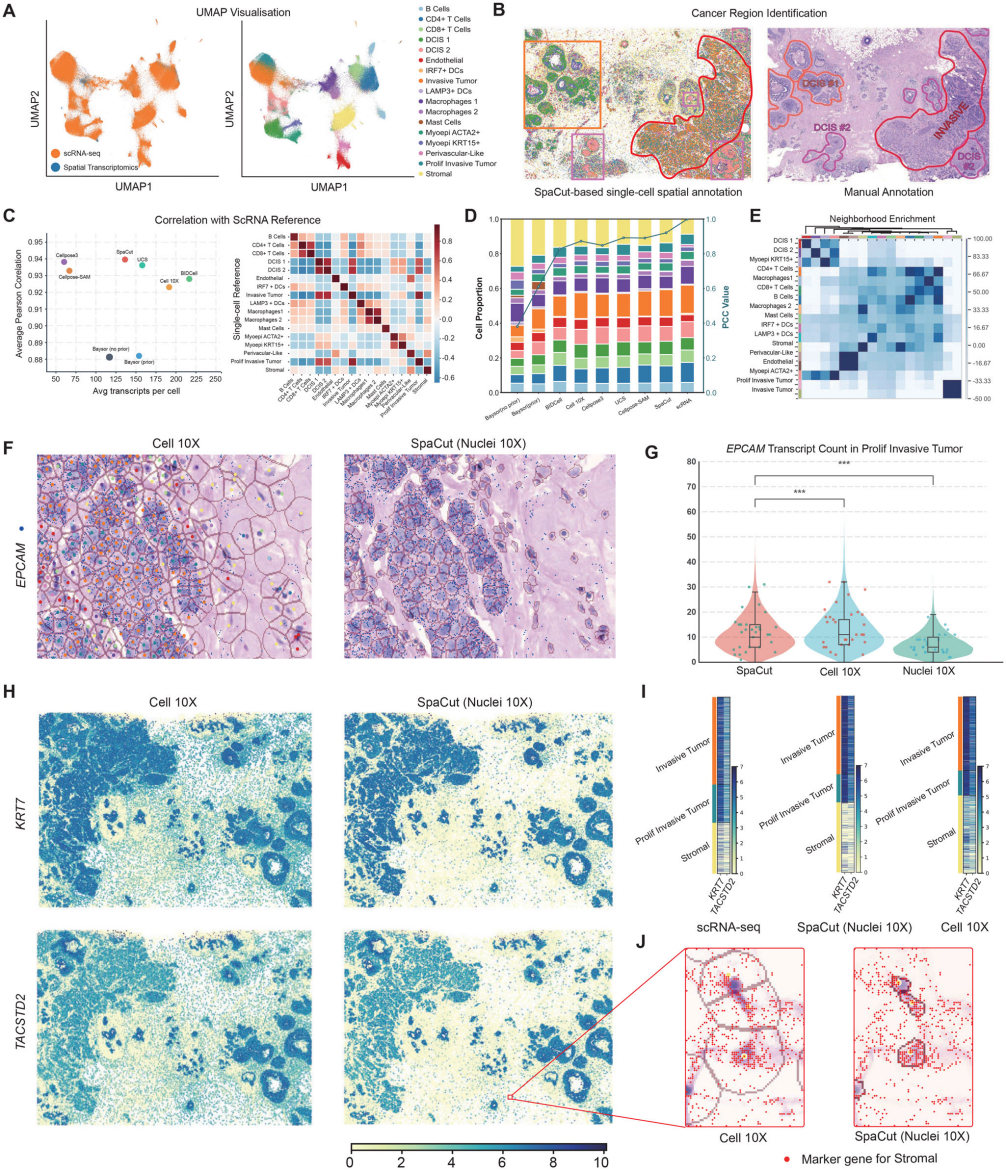
Overall performance and biological validation of SpaCut on the Xenium Human Breast Cancer dataset. (A) UMAP projection integrating ST data with scRNA‐seq reference. (B) Visual comparison between the computationally identified cancer regions (Left) and the annotated ground truth (Right). (C) Correlation with the scRNA‐seq reference. Method comparison by average Pearson correlation vs. average transcripts per cell (Left). Cell‐type Pearson correlation heatmap based on average gene expression (Right). (D) Descriptive comparison of cell‐type proportion concordance (PCC) across segmentation methods. (E) Neighborhood enrichment heatmap showing spatial cell–cell interactions. (F) Spatial visualization of the epithelial marker *EPCAM* with segmentation boundaries. (G) Violin plots quantifying *EPCAM* transcript capture sensitivity (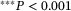). (H) Spatial distribution maps of tumor markers *KRT7* and *TACSTD2*. (I) Spatial visualization of tumor marker genes *KRT7* and *TACSTD2*, comparing their expression across different cell types for scRNA, SpaCut, and Cell 10×. (J) Zoom‐in views showing the spatial relationship between segmentation masks and stromal marker transcripts (red dots).

Qualitative evaluations further validated these quantitative results, confirming that SpaCut generates expression profiles highly congruent with the scRNA‐seq reference. As illustrated in Figure 6I, SpaCut improves this alignment with scRNA‐seq benchmarks by preserving accurate cell boundaries. This segmentation accuracy yields expression profiles that closely resemble the scRNA‐seq data, producing clearer spatial gene patterns for tumor marker genes like *KRT7* [33] and *TACSTD2* [34] (see Figure 6H). Additionally, the method facilitates the identification of marker genes by excluding contamination from extracellular stromal cells (Figure 6F). Importantly, the transcript counts recovered by SpaCut remain comparable to the oversized Cell 10X segmentation results (Figure 6G).

Finally, leveraging the precise cell typing enabled by SpaCut, we performed neighborhood enrichment analysis to explore cellular interactions (Figure 6E). This analysis uncovered biologically meaningful spatial organizations. Notably, SpaCut captured strong co‐localization between Proliferative Invasive Tumor and Invasive Tumor cells, as well as the close proximity between Myoepithelial ACTA2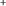 and Stromal populations, consistent with known epithelial–stromal interactions in breast cancer. Furthermore, immune‐related neighborhoods were clearly resolved: CD4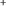 and CD8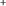 T cells showed significant enrichment near IRF7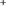 [35] and LAMP3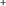 [36] dendritic cells. SpaCut remained consistent across breast cancer sections without fine‐tuning (Table S3 and Figures S8 and S9). Across the two test sections, annotation metrics changed by at most 0.0038, and the corresponding segmentation masks remained highly concordant, indicating stable transfer across sections. Under a common annotation and downstream‐analysis pipeline, SpaCut achieved competitive cell‐type annotation and the highest concordance of differential‐expression effect sizes (mean logFC PCC =0.9139). It also most closely reproduced the supervised neighborhood‐enrichment structure (PCC =0.9242; RMSE =1.1551), while its expression‐derived ligand–receptor signatures showed high reference concordance (PCC =0.9058). These results support strong overall biological consistency rather than uniform superiority across every endpoint (Tables S4– S7 and Figures S10–S13). Collectively, these findings highlight SpaCut's capacity to preserve biologically relevant spatial context, enabling the discovery of intercellular relationships critical for understanding the structural and functional complexity of tumor ecosystems.

### 2.6 Impact of Nuclei Segmentation Quality on SpaCut Performance

SpaCut relies on nuclei segmentation as it uses the nuclei as the foundational anchor for reconstructing complete cell boundaries in spatial transcriptomics. Variations in the quality of nuclei segmentation directly affect the accuracy of SpaCut's final cell segmentation results. To systematically evaluate this dependency, we conducted an ablation study using the Xenium Human Lung Cancer dataset, which provides official nuclei and cell segmentation annotations as ground truth. We compared SpaCut's performance across segmentation results obtained from a panel of representative image‐based methods spanning a broad range of segmentation quality from low to high.

As shown in (Figure 7A), the DAPI‐stained nuclei and corresponding masks highlight the quality gap between the official 10× nuclei masks (Nuclei 10×), which we use as reference annotations, and lower‐quality methods such as Watershed, which fail to produce reliable instance‐level nuclei segmentations. When these segmentation results were used as initialization inputs for SpaCut, their quality directly influenced the accuracy of the inferred cell boundaries. Using the official 10× nuclei input(Nuclei 10×), SpaCut generated cell boundaries that closely matched the Cell 10× reference, with accurate instance contours and minimal under‐ or over‐segmentation. Conversely, lower‐quality nuclei segmentations fail to adequately delineate nuclear boundaries, thereby impairing SpaCut's ability to infer accurate cell boundaries.

**FIGURE 7:**
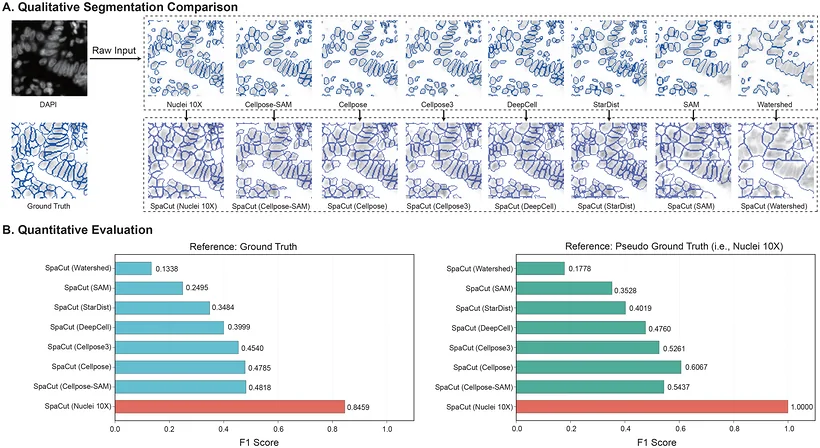
Evaluation of SpaCut using different nuclear segmentation inputs on the Xenium Human Lung Cancer dataset. (A) Qualitative segmentation comparison. The left column displays the raw DAPI image and Ground Truth annotations. The top row visualizes segmentation results generated by various image‐based methods (including 10× official nuclei segmentation, Cellpose‐SAM, Cellpose, Cellpose3, DeepCell, StarDist, SAM, and Watershed), which serve as inputs for SpaCut. The bottom row shows the corresponding refined segmentation outputs generated by SpaCut. (B) Quantitative evaluation. F1 scores of different methods. The evaluation is performed using two references: SpaCut initialized with 10× Nuclei as a pseudo ground truth (left) and true annotations (right).

To further quantify these observations, we evaluated the relationship between nuclei segmentation quality and SpaCut accuracy along two complementary dimensions. First, algorithmic consistency measures the internal stability of SpaCut outputs by comparing results from different nuclei segmentations against the Nuclei 10× benchmark (Figure 7B). Under this assessment, high‐quality segmentations such as Cellpose‐SAM and Cellpose achieved strong consistency (F1 = 0.54 and 0.61), while lower‐quality inputs such as SAM and Watershed showed substantial divergence (F1 = 0.35 and 0.18). Second, biological accuracy evaluates the alignment of SpaCut outputs with the official Cell 10× cell boundary annotations. Under this criterion, SpaCut achieved its highest accuracy when initialized with Nuclei 10× (F1 = 0.85), whereas inputs from less accurate segmentations resulted in a marked decline in performance.

Together, these results show that nuclei segmentation quality is a primary factor controlling SpaCut's segmentation accuracy. When nuclei are well segmented, their locations and extents provide reliable anchors for SpaCut to place cell boundaries in complex tissue regions. SpaCut refines the cell candidate associated with each supplied nuclear anchor and currently does not perform multi‐anchor merging or anchor‐free recovery. Consistently, instance F1 decreased monotonically as 10%–30% of anchors were removed, merged, or split, with missing anchors causing the largest reduction (from 0.8470 to 0.4775 at 30%). These results confirm that upstream nuclear‐mask quality directly constrains cell recovery (Table S8 and Figures S14 and S15).

### 2.7 Ablation Studies and Computational Performance

We evaluated the contributions of NGAF and GWA through component ablations on three Xenium datasets (Table S9). Within NGAF, removing spatial attention or the concatenation of the original and modulated nuclear softmasks reduced segmentation performance, indicating that both spatially selective aggregation and retention of the nuclear prior contribute to boundary refinement. Within GWA, removing the depthwise‐separable convolution and channel‐recalibration block (DSC_CA) also decreased AJI and F1. Replacing multi‐depth feature fusion with only the deepest feature map caused the largest single‐component reduction on Xenium Human Lung Cancer, lowering AJI and F1 from 0.7465 and 0.9395 to 0.6805 and 0.8871, respectively. Simultaneously removing spatial attention and DSC_CA further degraded performance, particularly on Xenium Mouse Colon. Overall, the complete model achieved the best AJI and F1 on all three datasets, supporting complementary roles for transcript‐feature encoding, multi‐depth aggregation, and nucleus‐guided fusion.

SpaCut also maintained a favorable balance between runtime and segmented‐cell count across the Xenium, Stereo‐seq, and CosMx benchmarks (Figure S16 and Tables S10–S12).

## 3 Discussion

In this study, we introduce SpaCut, a robust cell segmentation framework for subcellular spatial transcriptomics based on a deep transcript–morphology fusion approach. SpaCut integrates a Gene‐Wise Attention (GWA) Module with a Nuclei‐Guided Attentive Fusion (NGAF) module. The former encodes high‐dimensional gene expression data to capture spatial context and suppress noise, while the latter dynamically modulates nuclear priors to guide cell boundary expansion based on actual transcriptomic signals. Finally, a progressive training strategy is employed to synergize global foreground localization with precise instance‐level boundary refinement.

We evaluated the effectiveness of SpaCut on seven datasets from four representative platforms, including Xenium, CosMx, MERSCOPE, and Stereo‐seq. The experimental results show that SpaCut consistently achieves higher segmentation accuracy (F1 score) and instance‐level quality (AJI) than state‐of‐the‐art baselines across various tissues. Beyond these metrics, we validated the biological relevance of our method. Unlike image‐based approaches that rely solely on nuclei, SpaCut leverages gene expression to identify cytoplasmic regions, thereby recovering valid transcripts located outside the nuclear boundary. Furthermore, transcript subsampling experiments demonstrated the model's robustness. SpaCut maintained accurate cell structures even with only 20% of the original transcripts, confirming its ability to handle the signal sparsity and technical noise often found in spatial transcriptomics.

We conducted systematic ablation experiments to dissect the impact of both architectural components and input quality on segmentation outcomes. These studies revealed that the GWA and NGAF modules are mutually indispensable: the former is critical for decoding sparse transcriptomic signals via deep feature aggregation, while the latter is essential for transforming rigid nuclear priors into biologically accurate cell boundaries. Furthermore, we investigated the dependency on nuclear segmentation quality. Our analysis reveals that segmentation performance is significantly correlated with the accuracy of the initial nuclear anchors. While high‐quality nuclei inputs unlock the full potential of SpaCut for precise boundary refinement, suboptimal inputs lead to performance degradation. This observation underscores that reliable nuclear detection serves as a critical prerequisite for the subsequent transcript‐driven expansion. Collectively, these findings validate the efficacy of the dual‐module strategy while highlighting the importance of upstream data quality.

Future work will focus on reducing the dependence on nuclear anchors by incorporating explicit multi‐anchor merging and anchor‐free candidate recovery for tissues containing multinucleated cells or weak or missing nuclear signals. We will also explore confidence‐aware nuclear guidance and adaptive post‐processing to improve robustness when upstream nuclear masks are imperfect.

In summary, SpaCut offers an effective and unified solution for analyzing subcellular spatial transcriptomics data, outperforming state‐of‐the‐art methods in segmentation performance. By integrating transcriptomic and morphological information for robust boundary inference across heterogeneous platforms, SpaCut facilitates more accurate downstream analyzes of tissue heterogeneity and cellular interactions.

## 4 Methods

### 4.1 Dataset Description

We leveraged seven spatial transcriptomics datasets generated from multiple experimental platforms (Table 2). These datasets served as a foundation for quantitatively evaluating the performance of our cell segmentation framework across diverse tissue types and technological conditions.

**TABLE 2:** Summary of datasets used in this study.

Datasets	Platform	Cells	Genes	Total transcripts	Reference
Human Breast Cancer	Xenium	167,780	313	34,442,716	[37]
Human Lung Cancer	Xenium	162,254	377	10,605,483	[37]
Mouse Colon	Xenium	219,797	379	68,190,447	[37]
Human Pancreas	Xenium	140,702	377	7,170,423	[37]
Human Pancreas	CosMx SMI	48,951	18,946	64,960,677	[14]
Ovarian Cancer	MERSCOPE	71,381	500	32,054,444	[12]
Mouse Brain	Stereo‐seq	—	26,177	80,768,701	[15]

Specifically, the Human Breast Cancer dataset was produced using the Xenium platform, containing 167 780 cells profiled with 313 genes and a total of 34 442 716 transcripts. The Human Lung Cancer dataset, also from Xenium, includes 162 254 cells with 377 genes and 10 605 483 transcripts. For the Mouse Colon dataset, Xenium profiling yielded 219 797 cells, 379 genes, and 68 190 447 transcripts. Additionally, the Xenium Human Pancreas dataset comprises 140 702 cells with 377 genes and 7 170 423 transcripts.

Beyond Xenium data, we incorporated cross‐platform resources. The CosMx Human Pancreas dataset generated by CosMx SMI contains 48 951 cells and a substantially larger gene panel (18 946 genes), resulting in 64 960 677 transcripts. The Ovarian Cancer3 dataset was obtained from the MERSCOPE platform, encompassing 71 381 cells with 500 genes and 32 054 444 transcripts. Lastly, the Mouse Brain dataset was derived from Stereo‐seq, featuring 26 177 genes and a total of 80 768 701 transcripts.

Together, these datasets span a wide range of tissue origins, experimental scales, and sequencing resolutions, thereby providing a comprehensive foundation for validating our segmentation model's robustness and generalizability in spatial transcriptomics applications.

### 4.2 Data Preprocessing

Spatial transcriptomics datasets from multiple platforms were preprocessed to generate standardized gene expression maps and nuclear segmentation masks. Raw transcript coordinates were filtered based on quality scores (QV ≥ 20 for Xenium datasets) and mapped to a regular grid with platform‐specific resolutions (0.5 μm for Stereo‐seq and CosMx, 1 μm for Xenium and MERSCOPE).

For each dataset, we represent the spatial transcriptomics data as a multi‐channel gene expression map $G\in R^{H\times W\times N}$, where H and W represent spatial dimensions and N is the number of genes. Each spatial location (i,j) contains the transcript count for gene k as $G_{ijk}$. Nuclear segmentation masks $M\in R^{H\times W}$ were obtained either from platform‐provided segmentation (Xenium and MERSCOPE) or from DAPI staining using Cellpose and were spatially aligned with the gene‐expression maps.

For datasets with large gene panels, we compared highly variable genes selected with SCANPY [38] and genes ranked by mean expression at subset sizes of 500 and 1000. The top 500 expressed genes retained clear spatial structure with limited additional benefit from increasing the subset size and were therefore used to balance spatial information and computational cost (Figure S17).

Large‐scale datasets present unique challenges due to their spatial scale and heterogeneity. The Stereo‐seq Mouse Brain dataset encompasses an entire adult mouse brain slice (approximately 5.3 × 7.0 $mm^{2}$) captured at 0.5 μm resolution, resulting in transcriptomic maps significantly larger than standard imaging‐based platforms. Given this scale, applying uniform gene selection across the entire tissue may overlook regional variations in gene expression patterns. To address this limitation, we partitioned the dataset into smaller, computationally manageable patches of 1200×1200 pixels—adopting the identical patch size configuration used in SCS [29] to ensure methodological consistency and comparability. Individual models were trained for each patch to capture local transcriptomic characteristics and regional gene expression heterogeneity. For NanoString CosMx datasets, the platform's inherent field‐of‐view (FOV) structure was leveraged for model training. Each FOV was processed independently, with separate models trained to accommodate inter‐regional transcriptomic diversity across different tissue areas. This FOV‐specific approach ensures optimal performance while respecting the platform's natural data organization.

### 4.3 Gene‐Wise Attention (GWA) Module

The Gene‐Wise Attention (GWA) module encodes the joint spatial transcript map into feature channels for foreground localization. Given a transcript map G, we divide it into overlapping patches $\left\{G^{\left(p\right)}\right\}$ of size h×w (default 48×48), where $G^{\left(p\right)}\in R^{h\times w\times N}$ and N is the number of input genes. The input‐gene channels are first projected into a feature space, in which the subsequent channel‐recalibration operations are performed.

To initiate feature extraction, the high‐dimensional input patch is first compressed through three consecutive 1×1 convolutions to reduce channel dimensionality while preserving full spatial resolution, yielding an initial feature map $h_{0}\in R^{h\times w\times 64}$. The representation is then hierarchically refined through four encoding stages. In each stage, we first employ a Depthwise Separable Convolution (DSC). Formally, let $h_{i-1}$ be the input to the i‐th stage; the intermediate feature ${\tilde{h}}_{i}$ is obtained by decomposing the convolution into depthwise ($K_{dw}$) and pointwise ($K_{pw}$) operations:

(1) $${\tilde{h}}_{i}=K_{pw}\left(K_{dw}\left(h_{i-1}\right)\right)$$

This factorization significantly reduces computational cost while preserving fine spatial details essential for modeling subcellular transcript distributions.

Subsequently, global average pooling (GAP) and global max pooling (GMP) summarize the spatial responses of each feature channel. These pooled features are forwarded to a shared multi‐layer perceptron (MLP) to generate feature‐channel recalibration weights. Based on ${\tilde{h}}_{i}$, the weighted output $h_{i}$ is computed as:

(2) $$h_{i}={\tilde{h}}_{i}⊙\sigma \left(\mathrm{MLP}(\mathrm{GAP}\left({\tilde{h}}_{i}\right)+\mathrm{GMP}\left({\tilde{h}}_{i}\right))\right)$$

where σ is the sigmoid activation and ⊙ denotes element‐wise multiplication. This strategy recalibrates transcript‐derived features and suppresses uninformative responses. No spatial pooling or downsampling is used throughout these stages [39]; all feature maps preserve the original h×w resolution.

To integrate features from different depths, we aggregate the outputs from all encoding stages. We first concatenate the feature maps $\left\{h_{0},h_{1},h_{2},h_{3},h_{4}\right\}$ to construct a composite feature representation $F_{\text{agg}}$. To suppress redundancy and emphasize informative features, we re‐employ the weighting mechanism formulated in Equation(2), yielding the encoded features $F_{\text{enc}}$:

(3) $$F_{\text{enc}}=F_{\text{agg}}⊙\sigma \left(\mathrm{MLP}(\mathrm{GAP}\left(F_{\text{agg}}\right)+\mathrm{GMP}\left(F_{\text{agg}}\right))\right)$$

The three 1×1 convolutions perform channel projection and dimensionality reduction before attention is applied. The resulting feature‐channel weights are aggregated across encoding depths to form the encoded transcript representation $F_{\mathrm{enc}}$.

Finally, the classification head processes the encoded features $F_{\text{enc}}$ through four successive 1×1 convolutional layers followed by a softmax activation, yielding a prediction map $P_{\text{fg}}\in R^{h\times w\times 2}$. This output comprises two distinct channels: the first channel represents the probability that a given pixel belongs to the background, while the second channel represents the probability that the pixel is part of any cell. For subsequent calculations, we specifically denote ${\hat{p}}_{ij}$ as the predicted foreground probability for the pixel at spatial location (i,j).

### 4.4 Nucleus Softmask Generation

A critical component of SpaCut is the nucleus softmask, designed to transform discrete nuclei segmentations into continuous probability maps for spatial guidance. Starting with an instance‐level nuclei segmentation M, we partition the data into patches $\left\{M^{\left(p\right)}\right\}$ aligned with the spatial transcriptomics input. Each patch is structured as $M^{\left(p\right)}\in R^{h\times w\times n_{\text{cells}}}$, where $n_{\text{cells}}$ indicates the number of nuclei within that patch. Formally, we define $M_{c}\in R^{h\times w}$ (for $c=1,\cdots ,n_{\text{cells}}$) as the discrete binary mask corresponding specifically to the c‐th nucleus.

To transform the discrete binary mask $M_{c}$ into a continuous spatial prior, we apply a temperature‐scaled sigmoid function to its Signed Distance Transform (SDT):

(4) $${\hat{M}}_{c}\left(i,j\right)=\sigma \;\left(\frac{d_{\text{signed}}\left(M_{c},i,j\right)}{\tau }\right)$$

where $d_{\text{signed}}\left(\cdot \right)$ is the signed distance from pixel (i,j) to the nearest nuclear boundary (positive inside and negative outside), and τ controls transition smoothness (τ=5 by default). This yields ${\hat{M}}_{c}\in \left(0,1\right)$, which decays continuously from the nuclear interior toward the background (Figure S18).

### 4.5 Nuclei‐Guided Attentive Fusion (NGAF) Module

To explicitly incorporate Nucleus Softmask into gene representations, we design the Nuclei‐guided Attentive Fusion (NGAF) module. As illustrated in Stage 2 (Figure 1), this module fuses the encoded gene features $F_{\text{enc}}$ with the nucleus softmask ${\hat{M}}_{c}$. Note that the individual softmasks are stacked along the last dimension(corresponding to $n_{\text{cells}}$). Since all nuclei belong to the same patch, they share the same encoding features $F_{\text{enc}}$, which is broadcasted to match the number of nuclei.

For each nucleus c, a FiLM‐inspired projection head [40], denoted by $\phi_{\alpha }$ and implemented with three consecutive 1×1 convolutional layers with batch normalization and ReLU, maps $F_{\text{enc}}$ to a spatial coefficient map $\alpha =\phi_{\alpha }\left(F_{\text{enc}}\right)\in R^{h\times w}$. This coefficient modulates the corresponding nuclear softmask:

(5) $${\hat{M}}_{\mathrm{mod}}^{c}={\hat{M}}_{c}⊙\alpha$$

The original and modulated softmasks are retained and fused as $F_{\text{joint}}^{c}=\mathrm{Conv}\left(\mathrm{Concat}\left({\hat{M}}_{c},{\hat{M}}_{\mathrm{mod}}^{c}\right)\right)$. Average and maximum pooling along the channel axis are then concatenated and processed by a 5×5 convolution to obtain a spatial‐attention map. The refined output is computed as:

(6) $$F_{\text{out}}^{c}=F_{\text{joint}}^{c}⊙\sigma \;\left({\text{Conv}}_{5\times 5}\;\left(\text{Concat}(\text{AvgPool}\left(F_{\text{joint}}^{c}\right),\;\text{MaxPool}\left(F_{\text{joint}}^{c}\right))\right)\right)$$

To generate instance‐level predictions, we project the refined features $F_{\text{out}}^{c}$ via a 1×1 convolution into a dual‐channel probability map $P_{c}\in R^{H\times W\times 2}$ for the c‐th nucleus, where c∈{1,⋯,C} indexes the nuclei identified within the current patch. Let $P_{c}^{\text{bg}}$ and $P_{c}^{\text{cell}}$ denote the two channels of $P_{c}$, representing the predicted probabilities for the background and the c‐th cell, respectively. These probabilities are normalized via Softmax to satisfy:

(7) $$P_{c}^{\text{bg}}\left(i,j\right)+P_{c}^{\text{cell}}\left(i,j\right)=1,\;\forall \left(i,j\right).$$

Since multiple background probabilities are generated (one for each nucleus), they are averaged pixel‐wise to form a unified background probability ${\bar{P}}^{\text{bg}}$:

(8) $${\bar{P}}^{\text{bg}}\left(i,j\right)=\frac{1}{C}\sum_{c=1}^{C}P_{c}^{\text{bg}}\left(i,j\right)$$

The final segmentation result for a pixel is determined by selecting the highest probability among the background and each cell in the patch:

(9) $${\hat{y}}_{i,j}=\underset{k\in \{0,\cdots ,C\}}{arg max}\left({\bar{P}}^{\text{bg}}\left(i,j\right),\;P_{1}^{\text{cell}}\left(i,j\right),\;\cdots ,\;P_{C}^{\text{cell}}\left(i,j\right)\right),$$

where k=0 denotes the background class, and k>0 assigns the pixel to the k‐th cell.

### 4.6 Training Details: Progressive Training Strategy

#### 4.6.1 Stage 1: Training of the Gene‐Wise Attention (GWA) Module

The training objective for the Gene‐Wise Attention (GWA) Module starts by defining positive and negative samples. For positive samples (foreground), pixels located within nuclei regions ($M^{\left(p\right)}>0$) serve as ground truth, ensuring the model accurately identifies areas that belong to cells.

Defining negative samples (background) is more challenging due to the uncertainty in cytoplasmic boundaries. To address this, we employ an adaptive background filtering strategy. First, the nuclei segmentation mask is dilated using a morphological operator δ with a 10×10 kernel over four iterations:

(10) $$M_{\text{dilated}}^{\left(p\right)}=\delta^{\left(4\right)}\left(M^{\left(p\right)},10\right)$$

This dilation creates an exclusion zone around each nucleus that likely contains cytoplasm. Pixels outside this dilated region are candidates for background. However, not all such pixels are reliable negatives—some may contain diffuse transcripts from nearby cells. To further filter background candidates, we compute the average gene expression in the non‐dilated region:

(11) $${\bar{g}}_{\text{local}}=\frac{1}{|\Omega_{\text{non-dilated}}|}\sum_{\left(i,j\right)\in \Omega_{\text{non-dilated}}}\sum_{k=1}^{N}G_{ijk}^{\left(p\right)}$$

where $\Omega_{\text{non-dilated}}=\left\{\left(i,j\right):M_{\text{dilated},ij}^{\left(p\right)}=0\right\}$. Only pixels with expression below this local threshold are labeled as background:

(12) $$B_{ij}^{\left(p\right)}=\left\{\begin{array}{cc} 1, & \text{if}\;M_{\text{dilated},ij}^{\left(p\right)}=0\;\text{and}\;\sum_{k=1}^{N}G_{ijk}^{\left(p\right)}<{\bar{g}}_{\text{local}},\\ 0, & \text{otherwise.} \end{array}\right.$$

This formulation ensures that non‐cellular regions (i.e., the negative samples) are accurately classified as background. To prevent the model from learning ambiguous patterns, pixels within the uncertain zone (those that fall neither within the nucleus nor in the confident background) are explicitly excluded from the training objective. The final training set is thus defined as $\Omega_{\text{train}}=\left\{\left(i,j\right):M_{ij}^{\left(p\right)}>0\;\text{or}\;B_{ij}^{\left(p\right)}=1\right\}$.

The weighted cross‐entropy loss for a single patch is computed as:

(13) $$L_{\text{fg}}=-\sum_{\left(i,j\right)\in \Omega_{\text{train}}}\left[w_{1}M_{ij}^{\left(p\right)}\log{\hat{p}}_{ij}+w_{2}B_{ij}^{\left(p\right)}\log\left(1-{\hat{p}}_{ij}\right)\right]$$

where ${\hat{p}}_{ij}$ is the predicted foreground probability for pixel (i,j), and $w_{1}$, $w_{2}$ are the weighted parameters that balance the contribution of positive and negative samples. This selective training approach enables the model to learn robust foreground‐background separation while avoiding ambiguous cytoplasmic regions.

#### 4.6.2 Stage 2: Training of the Nuclei‐guided Attentive Fusion (NGAF) Module

In the second stage, the Nuclei‐guided Attentive Fusion (NGAF) Module is trained to refine cell boundaries with frozen encoded features $F_{\text{enc}}$. This freezing operation is critical: it prevents the cell‐specific training from corrupting the learned foreground features, ensuring stable and consistent boundary refinement across all cells.

For each cell c, positive samples are defined as pixels within the nucleus region ($M_{c,ij}^{\left(p\right)}=1$). Unlike the first stage, negative samples are now determined by the predictions of Stage 1: pixels with foreground probability below 0.5 (${\hat{p}}_{ij}<0.5$) are treated as confident background. Pixels in the uncertain zone ($0.5\leq {\hat{p}}_{ij}<1$ and outside the nucleus) are again excluded from training, allowing the model to focus on learning clear cell‐specific boundaries without interference from ambiguous regions.

The training region for cell c is defined as:

(14) $$\Omega_{\text{cell}}^{\left(c\right)}=\left\{\left(i,j\right):M_{c,ij}^{\left(p\right)}=1\;\text{or}\;{\hat{p}}_{ij}<0.5\right\}$$

The per‐cell loss is:

(15) $$L_{\text{cell}}^{\left(c\right)}=-\sum_{\left(i,j\right)\in \Omega_{\text{cell}}^{\left(c\right)}}\left[w_{3}M_{c,ij}^{\left(p\right)}\log P_{c}^{\text{cell}}\left(i,j\right)+w_{4}\left(1-M_{c,ij}^{\left(p\right)}\right)\log P_{c}^{\text{bg}}\left(i,j\right)\right]$$

where $P_{c}^{\text{cell}}\left(i,j\right)$ and $P_{c}^{\text{bg}}\left(i,j\right)$ denote the predicted probabilities for the foreground (cell c) and background at pixel (i,j). The parameters $w_{3}$ and $w_{4}$ serve as weighting coefficients to balance the contribution of the positive and negative samples. The final loss for a patch containing C cells is the average over all cells:

(16) $$L_{\text{cell}}=\frac{1}{C}\sum_{c=1}^{C}L_{\text{cell}}^{\left(c\right)}$$

In this formulation, $M_{c,ij}^{\left(p\right)}$ is the binary nucleus mask for cell c. This weighted cross‐entropy loss optimizes robust instance‐level separation by accurately distinguishing between the cell interior and predicted background. Crucially, it exploits the stable foreground features learned in Stage 1 to achieve precise boundary refinement.

This progressive optimization strategy sequentially establishes coarse foreground localization and subsequently refines cell boundaries using frozen features, thereby improving segmentation precision by leveraging coarse localization cues to guide accurate cell segmentation.

### 4.7 Evaluation Metrics

Model performance was assessed using F1 score and Aggregated Jaccard Index (AJI) for instance‐level segmentation quality. To provide a comprehensive evaluation, the F1 score was computed at the instance level across various matching thresholds.

Instance‐level F1 Score: Instead of pixel‐level evaluation, we measure the detection and segmentation accuracy of individual cells. The spatial overlap between a predicted cell mask (P) and a ground truth cell mask (G) is quantified by the Intersection over Union (IoU):

(17) $$\text{IoU}\left(P,G\right)=\frac{\left|P\cap G\right|}{\left|P\cup G\right|}$$

Given an IoU threshold τ, a predicted cell is considered a True Positive (TP) if its maximum IoU with a ground truth cell satisfies IoU(P,G)≥τ. Predicted cells that fail to meet this threshold are counted as False Positives (FP), and ground truth cells without a valid matching prediction are counted as False Negatives (FN). Based on these instance‐level counts, Precision, Recall, and the F1 score are calculated as:

(18) $$\text{Precision}=\frac{TP}{TP+FP}$$

(19) $$\text{Recall}=\frac{TP}{TP+FN}$$

(20) $$\text{F1}@\tau =2\cdot \frac{\text{Precision}\times \text{Recall}}{\text{Precision}+\text{Recall}}=\frac{2\cdot TP}{2\cdot TP+FP+FN}$$

By varying the matching threshold τ (e.g., from 0.1 to 0.9), we obtain an F1‐IoU curve that reflects the model's robustness under increasingly strict segmentation criteria.

Aggregated Jaccard Index (AJI): AJI evaluates instance‐level segmentation quality by measuring overlap between predicted and ground truth cell instances:

(21) $$\text{AJI}=\frac{\sum_{i=1}^{N}\left|G_{i}\cap P_{\sigma (i)}\right|}{\sum_{i=1}^{N}|G_{i}\cup P_{\sigma (i)}|+\sum_{j\in U}\left|P_{j}\right|}$$

where $G_{i}$ represents the i‐th ground truth cell instance, $P_{j}$ denotes the j‐th predicted cell instance, σ(i) is the predicted instance with maximum overlap to $G_{i}$, N is the number of ground truth instances, and U is the set of unmatched predicted instances. The AJI ranges from 0 to 1, with higher values indicating better instance‐level segmentation performance.

### 4.8 Experiment Settings

Experiments were conducted on a single NVIDIA RTX 3090 GPU with PyTorch 2.0.1 [41] and Python 3.9. The SpaCut framework employed a progressive training strategy optimized by Adam with a learning rate of $10^{-4}$. Specifically, the GWA Module was trained for 10 epochs with a batch size of 128, while the NGAF module was subsequently trained for 10 epochs with a batch size of 1 and frozen encoder features. Loss weights $w_{1},w_{2},w_{3},w_{4}$ were set to 1 to ensure balanced supervision. Data augmentation included random discrete rotation (0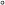, 90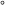, 180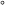, 270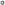) and vertical flipping. All experiments were independently repeated five times with different random seeds, and the final results were reported as the mean ± standard deviation across runs. SpaCut uses the same post‐processing pipeline as BIDCell [30], consisting of several standard morphological operations. Standard morphological post‐processing may remove fragmented regions but can also modify correctly delineated boundary pixels. Its effect is therefore condition‐dependent, and it is treated as an optional cleanup step rather than a procedure expected to improve all quantitative metrics. For downstream single‐cell processing, spatial neighborhood analysis, and visualization, we utilized Scanpy [38] and Squidpy [42].

### 4.9 Baseline Methods

To benchmark SpaCut across complementary segmentation paradigms, we selected eleven representative methods spanning integrative, transcript‐based, and image‐based approaches: BIDCell [30], Proseg [27], SCS [29], UCS [31], Baysor [26], DeepCell [22], SAM [21], Cellpose [18], Cellpose‐SAM [20], StarDist [19], and MEDIAR [23]. Their algorithmic principles and execution configurations are summarized below.

**BIDCell** [30]: BIDCell is a self‐supervised framework for cell segmentation in subcellular spatial transcriptomics. It integrates biologically informed loss functions with single‐cell RNA‐seq references to model relationships between gene expression and cell morphology. We used the documented Xenium configuration.

**Proseg** [27]: Proseg is a probabilistic segmentation method that adapts the Cellular Potts model within a Metropolis–Hastings sampling framework. It infers cell boundaries by optimizing the likelihood of transcript distributions and includes transcript repositioning to reduce spurious co‐expression. The default configuration was used.

**SCS** [29]: SCS employs a transformer‐based neural network to combine gene‐expression profiles with high‐resolution imaging information. It learns the relative position of each spot to its corresponding cell center and groups spots through gradient‐flow tracking. We used the default parameters provided by the authors.

**UCS** [31]: UCS uses a two‐stage nucleus‐anchored framework. Its first network extracts transcript‐derived foreground features, and its second network combines these features with distance‐based nuclear softmasks for per‐anchor cell prediction. The model was run using the documented implementation and dataset‐specific settings.

**Baysor** [26]: Baysor uses a Bayesian mixture model that accounts for spatial morphology and transcriptional composition when assigning molecules to cells. Both spatial‐prior and no‐prior configurations were evaluated using the documented settings.

**DeepCell** [22]: DeepCell is a deep‐learning framework for whole‐cell and nuclear segmentation in tissue images. It uses a ResNet50‐based backbone with a feature‐pyramid architecture trained on TissueNet. We used the official pretrained model.

**SAM** [21]: The Segment Anything Model uses a Vision Transformer trained on the SA‐1B dataset and provides promptable zero‐shot segmentation. We used the official pretrained weights in the image‐based comparison.

**Cellpose** [18]: Cellpose predicts spatial gradient‐flow fields and forms cellular instances by grouping pixels that converge to common sinks. We used the official pretrained weights.

**Cellpose‐SAM** [20]: Cellpose‐SAM combines the Cellpose flow‐field formulation with a SAM‐based image encoder to improve generalization across microscopy images. We used the official pretrained model without architecture‐specific retraining.

**StarDist** [19]: StarDist represents nuclei as star‐convex polygons and predicts radial distances to their boundaries together with object probabilities. We used the official pretrained weights.

**MEDIAR** [23]: MEDIAR is a transformer‐based framework for cell‐instance segmentation in multimodal microscopy images. We evaluated it with the official pretrained model.

### Author Contributions

W.M. and F.Z. conceived and supervised the project. Y.L., F.Z. and W.M. developed and implemented the SpaCut algorithm. All authors collaborated on methodological discussions, validation studies, and manuscript preparation. All authors read and approved the final manuscript. Y.L. and F.Z. contributed equally to this work.

### Funding

The work was supported in part by the National Natural Science Foundation of China (Grant Nos. 62262069 and 62662067), the Scientific Research Fund Project of Yunnan Education Department (Grant No. 2026J0841), and Yunnan Fundamental Research Projects (Grant No. 202601CI070001).

## Ethics Statement

Not applicable. All datasets used in this study are publicly available.

## Consent

The authors have nothing to report.

## Conflicts of Interest

The authors declare no conflicts of interest.

## Supporting information


**Supporting File**: advs78423‐sup‐0001‐SuppMat.pdf.

## Data Availability

All datasets analyzed in this work are publicly available from the following sources: Xenium Human Breast Cancer (Replicate 1 and Replicate 2) and scRNA reference: https://www.10xgenomics.com/products/xenium‐in‐situ/preview‐dataset‐human‐breast Xenium Human Lung Cancer: https://www.10xgenomics.com/datasets/preview‐data‐ffpe‐human‐lung‐cancer‐with‐xenium‐multimodal‐cell‐segmentation‐1‐standard Xenium Mouse Colon: https://www.10xgenomics.com/datasets/fresh‐frozen‐mouse‐colon‐with‐xenium‐multimodal‐cell‐segmentation‐1‐standard Xenium Human Pancreas: https://www.10xgenomics.com/datasets/ffpe‐human‐pancreas‐with‐xenium‐multimodal‐cell‐segmentation‐1‐standard NanoString CosMx Human Pancreas: https://nanostring.com/products/cosmx‐spatial‐molecular‐imager/ffpe‐dataset/cosmx‐smi‐human‐pancreas‐ffpe‐dataset/ Vizgen MERSCOPE Ovarian Cancer3: https://info.vizgen.com/ffpeshowcase Stereo‐seq Mouse Brain: https://db.cngb.org/stomics/mosta/download/ In addition, the curated data and annotations generated in this study have been deposited on Zenodo and are available at https://zenodo.org/uploads/18012899.
