# Supplementary material for: SpaCut: Transcript‐Aware Morphology Fusion for Robust Cell Segmentation in Subcellular Spatial Transcriptomics
Source: Adv Sci (Weinh). 2026 Oct 29:e78423. Online ahead of print. doi: 10.1002/advs.78423 (PMC13720276; doi:10.1002/advs.78423)
Supplement: Supplementary file 1 [file ADVS-9999-e78423-s001.pdf]

Supplementary Material:

SpaCut: Transcript-aware Morphology Fusion for Robust Cell Segmentation in Subcellular Spatial Transcriptomics

Contents

1 Supplementary Tables **2**

2 Supplementary Figures **6**

1 Supplementary Tables

Table S1: Transcript-assignment results across three Xenium datasets. Rec., correct cytoplasmic recovery; Nei., neighboring-cell contamination; Ext., extracellular contamination. **Bold** indicates the best correct-recovery and assignment-F1 result within each dataset.

Metric	SpaCut	UCS	Baysor (prior)	Baysor (no prior)	Cellpose	Cellpose3	Cellpose-SAM	DeepCell	MEDIAR	SAM	StarDist
**Lung cancer**											
Rec. ↑	**0.7324**	0.3738	0.6622	0.4947	0.2568	0.2002	0.3180	0.2734	0.2049	0.2306	0.2440
Nei. ↓	**0.0992**	0.1947	0.1364	0.3821	0.1419	0.1177	0.1625	0.1487	0.1196	0.2264	0.1415
Ext. ↓	**0.0973**	0.1089	0.1155	0.1168	0.0271	0.0227	0.0325	0.0285	0.0225	0.0302	0.0277
F1 ↑	**0.8371**	0.6962	0.7545	0.5175	0.6358	0.5923	0.6672	0.6451	0.5955	0.5476	0.6145
**Human pancreas**											
Rec. ↑	**0.6964**	0.4634	0.5337	0.4526	0.2567	0.2144	0.3208	0.2830	0.2235	0.2494	0.2471
Nei. ↓	**0.1320**	0.2131	0.2621	0.3878	0.2371	0.2199	0.2752	0.2358	0.2209	0.2828	0.2194
Ext. ↓	**0.1584**	0.2094	0.2135	0.2069	0.1081	0.1033	0.1129	0.1133	0.1045	0.1105	0.1074
F1 ↑	**0.7619**	0.6227	0.5430	0.4309	0.4257	0.3808	0.4734	0.4483	0.3901	0.4038	0.4151
**Mouse colon**											
Rec. ↑	**0.7288**	0.4769	0.5957	0.4149	0.2778	0.2193	0.3070	0.2814	0.2305	0.1869	0.2618
Nei. ↓	**0.1084**	0.1874	0.2638	0.4462	0.2670	0.2346	0.2813	0.2558	0.2351	0.3498	0.2532
Ext. ↓	**0.1686**	0.2231	0.2370	0.2364	0.0956	0.0912	0.0923	0.0982	0.0903	0.1142	0.0948
F1 ↑	**0.7825**	0.6423	0.5573	0.3564	0.4767	0.4286	0.4964	0.4816	0.4415	0.3296	0.4593

Table S2: Controls for the boundary-learning signal on Xenium Human Lung Cancer.

Condition	F1 ↑	AJI ↑
w/ full SpaCut input, w/o post-processing	0.8638	0.6263
w/ shuffled transcripts, w/o post-processing	0.7389	0.5545
w/o transcripts, w/o post-processing	0.7032	0.5437
w/o transcript condition, w/o post-processing	0.6643	0.5251
Fixed dilation (5 pixels)	0.8352	0.6205
Analytic softmask	0.8264	0.6152
Nuclei only	0.6611	0.5235
w/ full SpaCut input, w/ post-processing	0.8470	0.6178
w/ shuffled transcripts, w/ post-processing	0.7471	0.5545
w/o transcripts, w/ post-processing	0.7200	0.5512
w/o transcript condition, w/ post-processing	0.6841	0.5339

Table S3: Bidirectional cross-replicate annotation performance of SpaCut.

Training section	Test section	Setting	Accuracy	Macro-F1	MCC	Evaluated cells
Section 1	Section 1	Within	0.7342	0.6738	0.7116	157,953
Section 2	Section 1	Cross	0.7349	0.6750	0.7119	157,930
Section 2	Section 2	Within	0.6166	0.5816	0.5765	114,388
Section 1	Section 2	Cross	0.6129	0.5839	0.5741	114,397

Table S4: Cell-type annotation consistency under the common scVI/scANVI pipeline. **Bold** indicates the best result for each primary performance metric.

Method	Match	Coverage ↑	Accuracy ↑	Macro-F1 ↑	Cohen's κ ↑	Segmented-cells
SpaCut	Direct	0.9528	**0.7342**	**0.6738**	**0.7025**	157,953
UCS	Direct	**0.9529**	0.7325	0.6733	0.7010	157,936
BIDCell	MNN	0.9353	0.6865	0.6192	0.6499	100,845
Baysor (prior)	MNN	0.7270	0.7122	0.6105	0.6778	154,246
Baysor (no prior)	MNN	0.4435	0.6395	0.5516	0.5940	112,459
Cellpose3	MNN	0.8565	0.6879	0.6131	0.6500	129,379
Cellpose-SAM	MNN	0.8800	0.6898	0.6079	0.6526	130,520

Table S5: Differential-expression consistency with the tissue-matched single-cell reference. **Bold** indicates the best result for each metric.

Method	Mean marker Jaccard ↑	Mean logFC PCC ↑
SpaCut	0.6607	**0.9139**
UCS	0.6686	0.9098
BIDCell	0.6528	0.9040
Baysor (prior)	0.5843	0.8471
Baysor (no prior)	0.5677	0.8192
Cellpose3	**0.6704**	0.9100
Cellpose-SAM	0.6622	0.9030

Table S6: Neighborhood-enrichment consistency with the supervised spatial reference. **Bold** indicates the best result for each metric.

Method	PCC ↑	RMSE ↓
SpaCut	**0.9242**	**1.1551**
UCS	0.9070	1.2359
BIDCell	0.9228	1.1972
Baysor (prior)	0.7854	1.8536
Baysor (no prior)	0.6187	2.4763
Cellpose3	0.8859	1.6052
Cellpose-SAM	0.8889	1.6267

Table S7: Ligand–receptor communication-signature consistency with the matched reference. **Bold** indicates the best result for each metric.

	Expression-derived		Spatially weighted	
Method	PCC ↑	RMSE ↓	PCC ↑	RMSE ↓
SpaCut	**0.9058**	**11.4551**	0.9116	1.9762
UCS	0.8948	12.1597	0.9150	1.9382
BIDCell	0.8936	13.0763	**0.9226**	**1.8440**
Baysor (prior)	0.7269	18.3860	0.7639	3.0677
Baysor (no prior)	0.6455	20.7932	0.7414	3.2267
Cellpose3	0.8429	15.3546	0.8700	2.5339
Cellpose-SAM	0.7764	17.7478	0.7404	3.3144

Table S8: Sensitivity to controlled nuclear-anchor errors on Xenium Human Lung Cancer.

Perturbation	Level	Affected anchors	F1 ↑	AJI ↑	Cell recall ↑
Official nuclei		0	0.8470	0.6178	0.8321
Missing	10%	15,661	0.7352	0.5210	0.6868
Missing	20%	31,322	0.6097	0.4400	0.5401
Missing	30%	46,984	0.4775	0.3703	0.3999
Merged	10%	15,662	0.7943	0.5757	0.7612
Merged	20%	31,322	0.7399	0.5396	0.6912
Merged	30%	46,984	0.6813	0.5070	0.6200
Split	10%	15,661	0.7449	0.5767	0.7677
Split	20%	31,322	0.6498	0.5369	0.7011
Split	30%	46,984	0.5626	0.4984	0.6342

Table S9: Ablation studies on NGAF and GWA modules across three Xenium datasets. **Bold** indicates the best result for each metric.

	Xenium Human Lung Cancer		Xenium FFPE Human Pancreas		Xenium Mouse Colon	
**Method**	**AJI**	**F1 Score**	**AJI**	**F1 Score**	**AJI**	**F1 Score**
w/o spatial attention (NGAF)	0.7363	0.9236	0.7404	0.8859	0.6013	0.8834
w/o softmask concatenation (NGAF)	0.7258	0.9284	0.7437	0.8913	0.5934	0.8820
w/o DSC_CA (GWA)	0.7284	0.9148	0.7351	0.8764	0.5938	0.8745
w/o multi-depth fusion (GWA)	0.6805	0.8871	0.7351	0.8652	0.5910	0.8865
w/o spatial attention and DSC_CA	0.7228	0.9107	0.7384	0.8804	0.5842	0.8621
**w/ all modules (SpaCut)**	**0.7465**	**0.9395**	**0.7451**	**0.8920**	**0.6151**	**0.8980**

Table S10: Runtime (in seconds) and Cell Count on **Stereo seq one patch**

	SCS	SpaCut	Cellpose	UCS	Cellpose-SAM
Runtime (s)	8126.18	435	10.8	418	16.8
Cell Count	875	1133	950	1120	1017

Table S11: Runtime (in seconds) and Cell Count on **CosMx Pancreas fov51**

	Proseg	SpaCut	Cellpose	UCS	Cellpose-SAM
Runtime (s)	763	532	24.94	688	29.8
Cell Count	2540	2514	2667	2520	2679

Table S12: Cell Count on three Xenium datasets

Method	Human Lung Cancer	Human Pancreas	Mouse Colon
StarDist	214235	176225	356014
DeepCell	189276	169900	280116
Cell 10X (GT)	162248	140701	219795
MEDIAR	160532	144579	221992
Cellpose3	158028	137412	214610
Nuclei 10X	156612	136520	206898
SpaCut (Ours)	156539	136446	206808
Baysor (prior)	155139	139574	213335
UCS	154574	134729	205090
Cellpose	150070	135886	201714
Cellpose-SAM	148870	136360	199413
SAM	125916	128153	114190
Baysor (no prior)	110508	98823	107452

2 Supplementary Figures

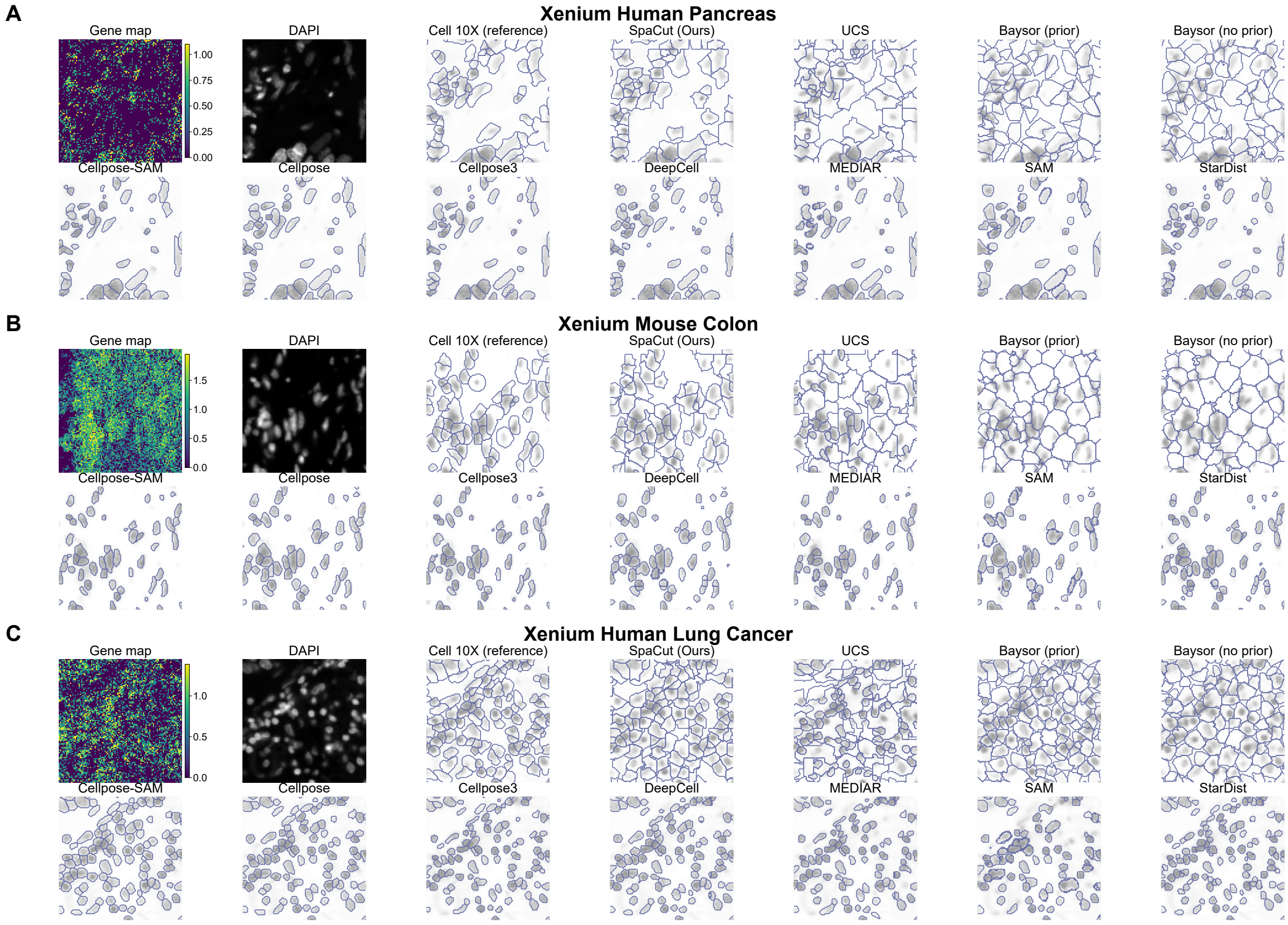

Figure S1: Refined qualitative comparison on three Xenium datasets. (A) Xenium Human Pancreas, (B) Xenium Mouse Colon, and (C) Xenium Human Lung Cancer. The first row shows the total-expression gene map, DAPI image, Cell 10X reference, SpaCut, UCS, and Baysor; the second row shows the image-based baselines. All panels within each dataset show the same field of view and use consistent visualization settings.

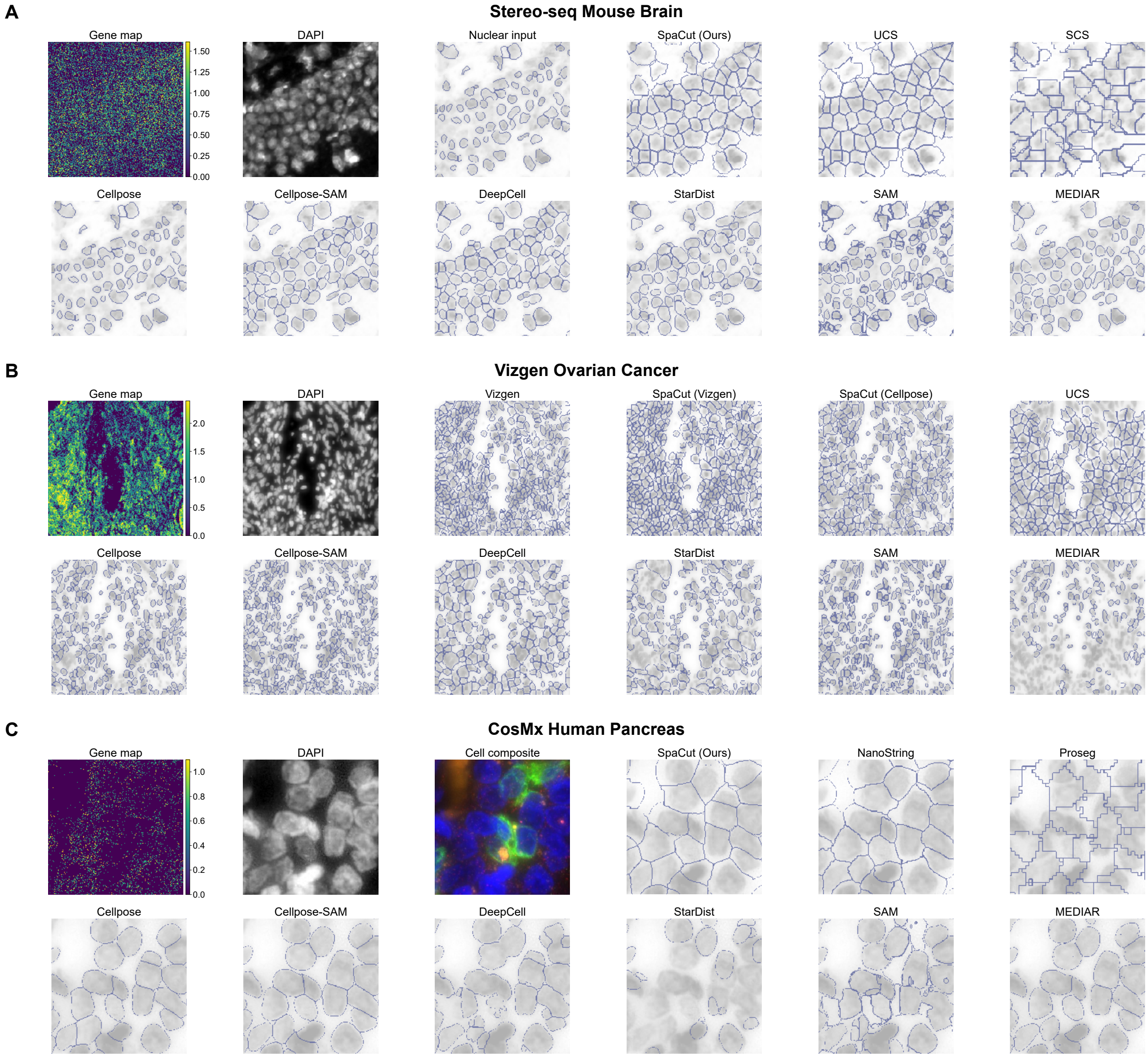

Figure S2: Expanded cross-platform same-region qualitative comparisons. (A) Stereo-seq Mouse Brain, (B) Vizgen Ovarian Cancer, and (C) CosMx Human Pancreas. For each dataset, the upper row shows the gene map, microscopy input, SpaCut, and platform-specific comparison methods; the lower row shows the common image-based baselines Cellpose, Cellpose-SAM, DeepCell, StarDist, SAM, and MEDIAR. All panels within each dataset display the identical selected field of view. Existing spatially aligned Cellpose and Cellpose-SAM predictions were reused; newly run image-based methods were inferred with surrounding image context and then cropped back to the displayed field to reduce edge effects. This figure is qualitative; no evaluation metric is computed from the selected fields.

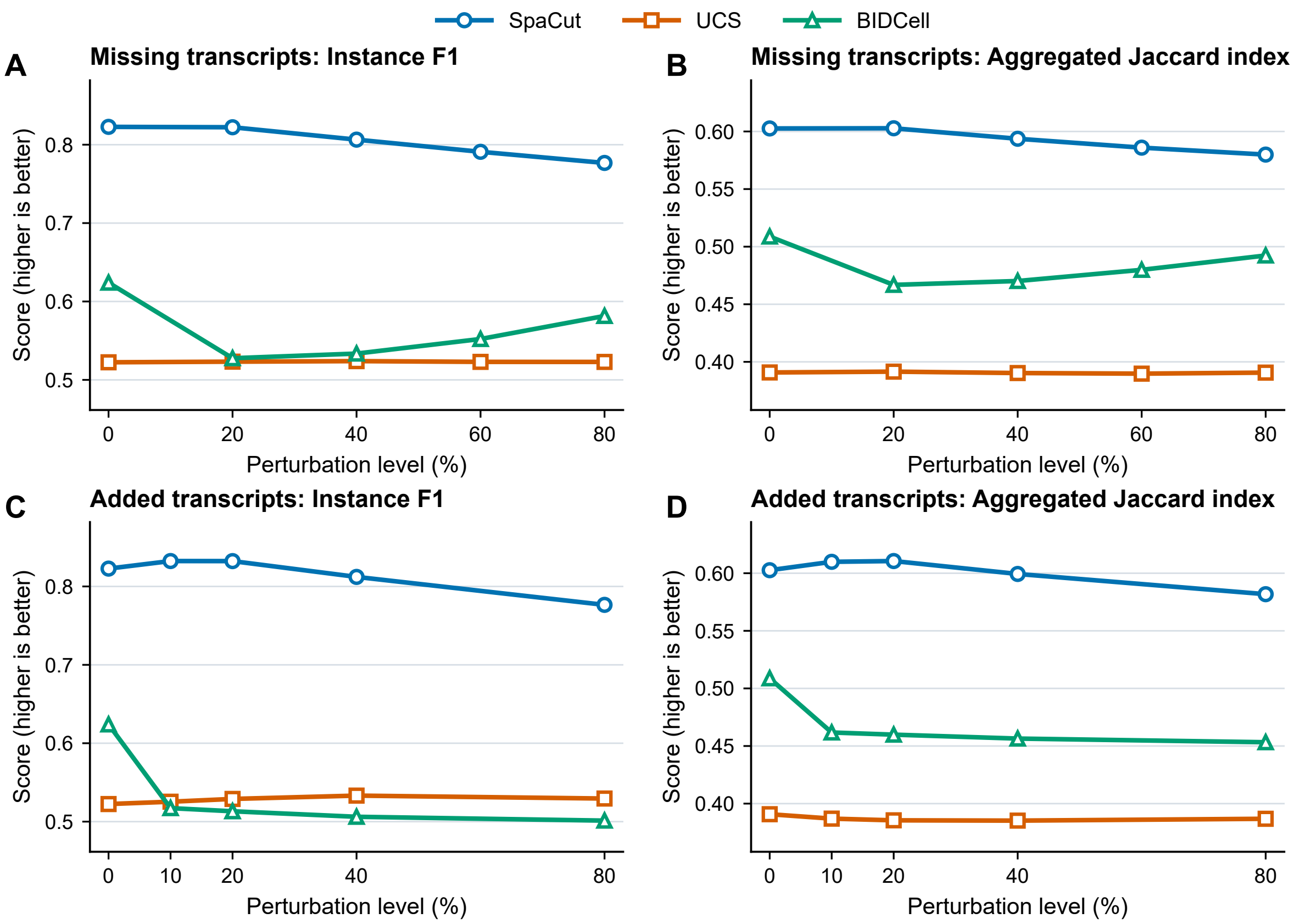

Figure S3: Quantitative robustness under transcript perturbations on Xenium Human Lung Cancer. Instance F1 at an IoU threshold of 0.5 and aggregated Jaccard index (AJI) are reported for SpaCut, UCS, and BIDCell under increasing transcript-missing rates (A, B) and added-background-transcript rates (C, D). Higher values indicate better segmentation agreement with the Cell 10X reference.

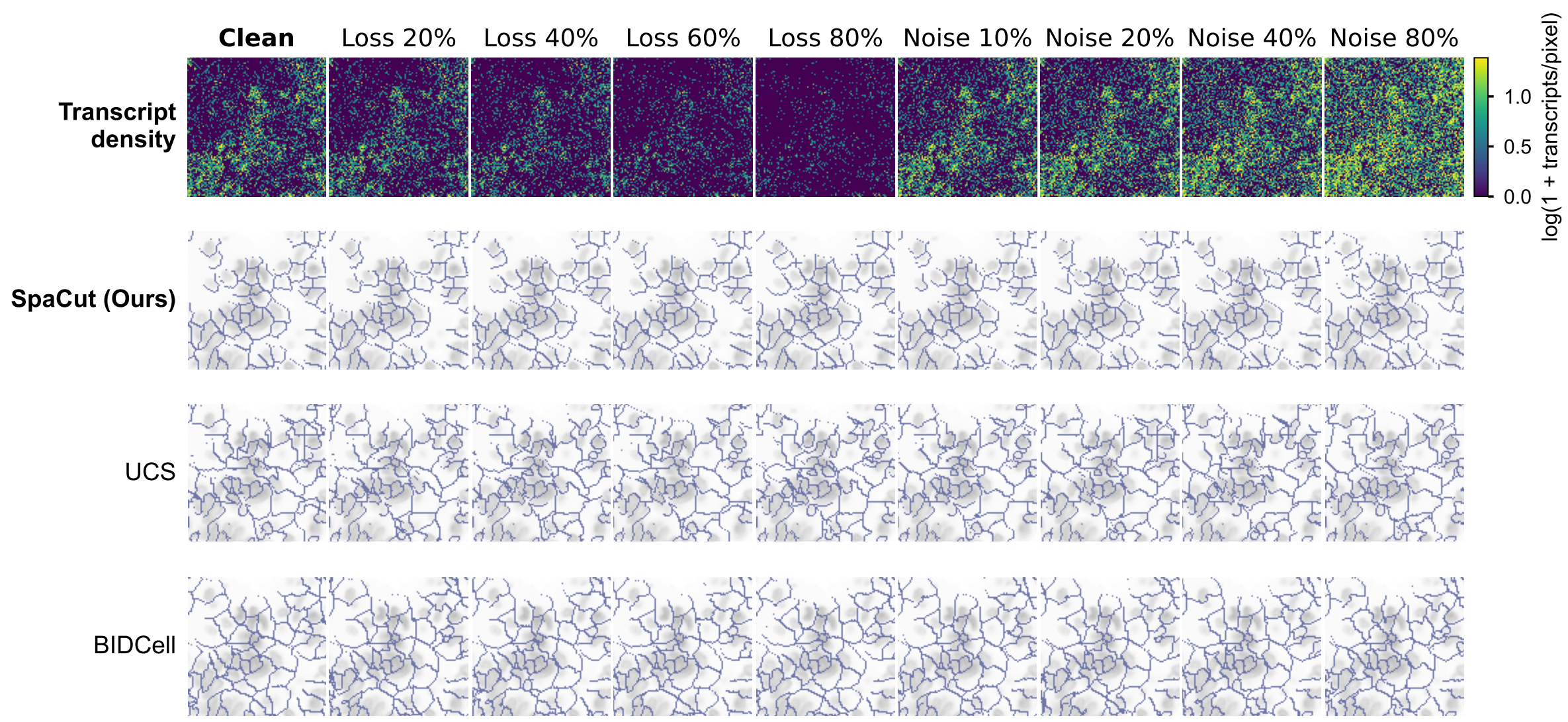

Figure S4: Qualitative robustness to transcript loss and added background transcripts on Xenium Human Lung Cancer. The complete perturbation series comprises the clean input, transcript loss at 20%, 40%, 60%, and 80%, and added background transcripts at 10%, 20%, 40%, and 80%. Transcript-density maps share a clean-input-anchored intensity scale. SpaCut, UCS, and BIDCell boundaries are shown on the same lightly rendered DAPI image and in the same fixed field of view.

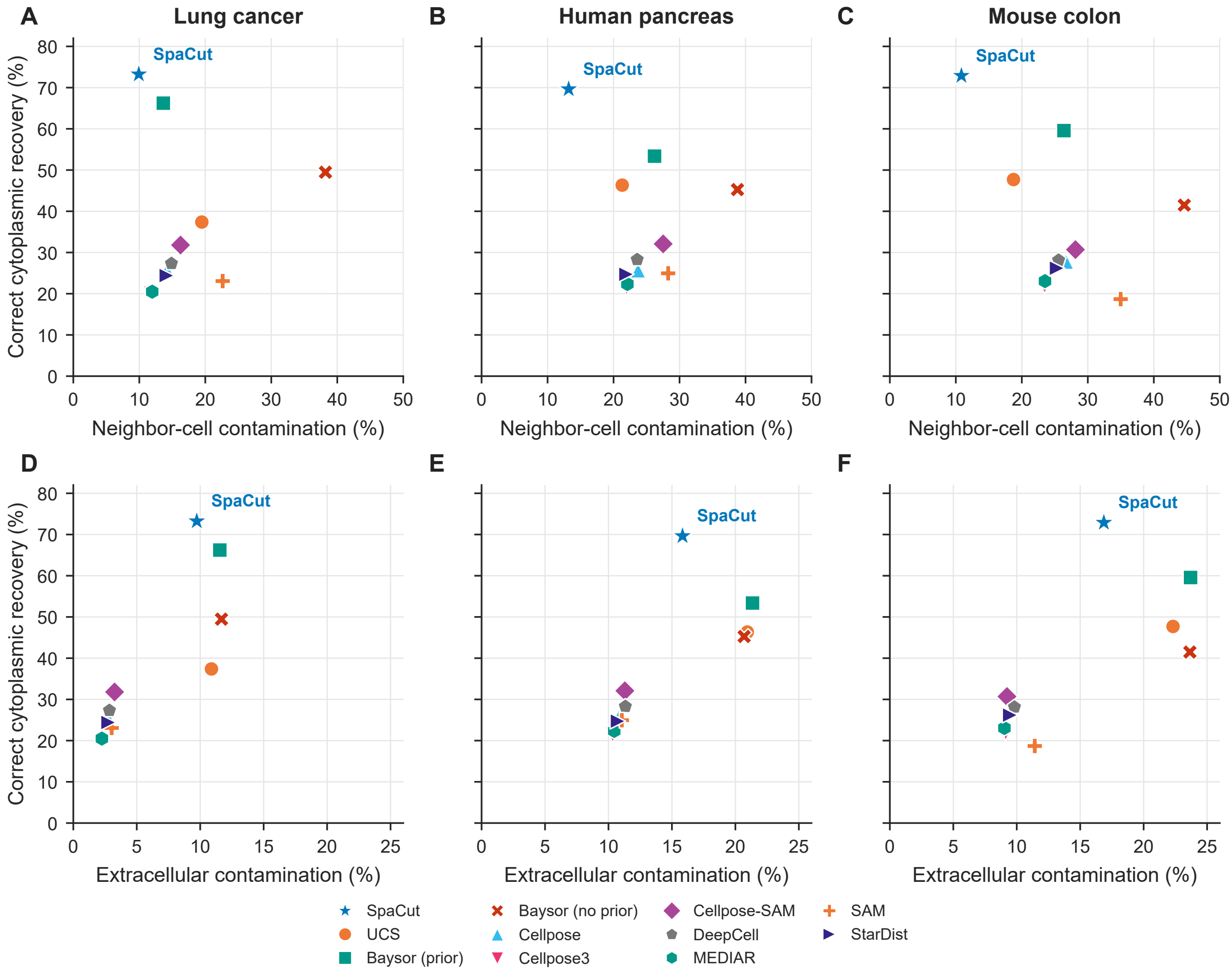

Figure S5: Correct cytoplasmic recovery versus contamination across all completed baselines. (A–C) Correct cytoplasmic recovery versus neighboring-cell contamination in Xenium Human Lung Cancer, Human Pancreas, and Mouse Colon. (D–F) Correct cytoplasmic recovery versus extracellular contamination in the same datasets. Each method is identified by a consistent color–marker combination, with SpaCut highlighted by a blue star. Movement toward the upper left indicates recovery of more correct cytoplasmic transcripts with less contamination. Cell 10X and Nuclei 10X are reference and nucleus-only controls, respectively, and are not ranked as segmentation baselines in this figure.

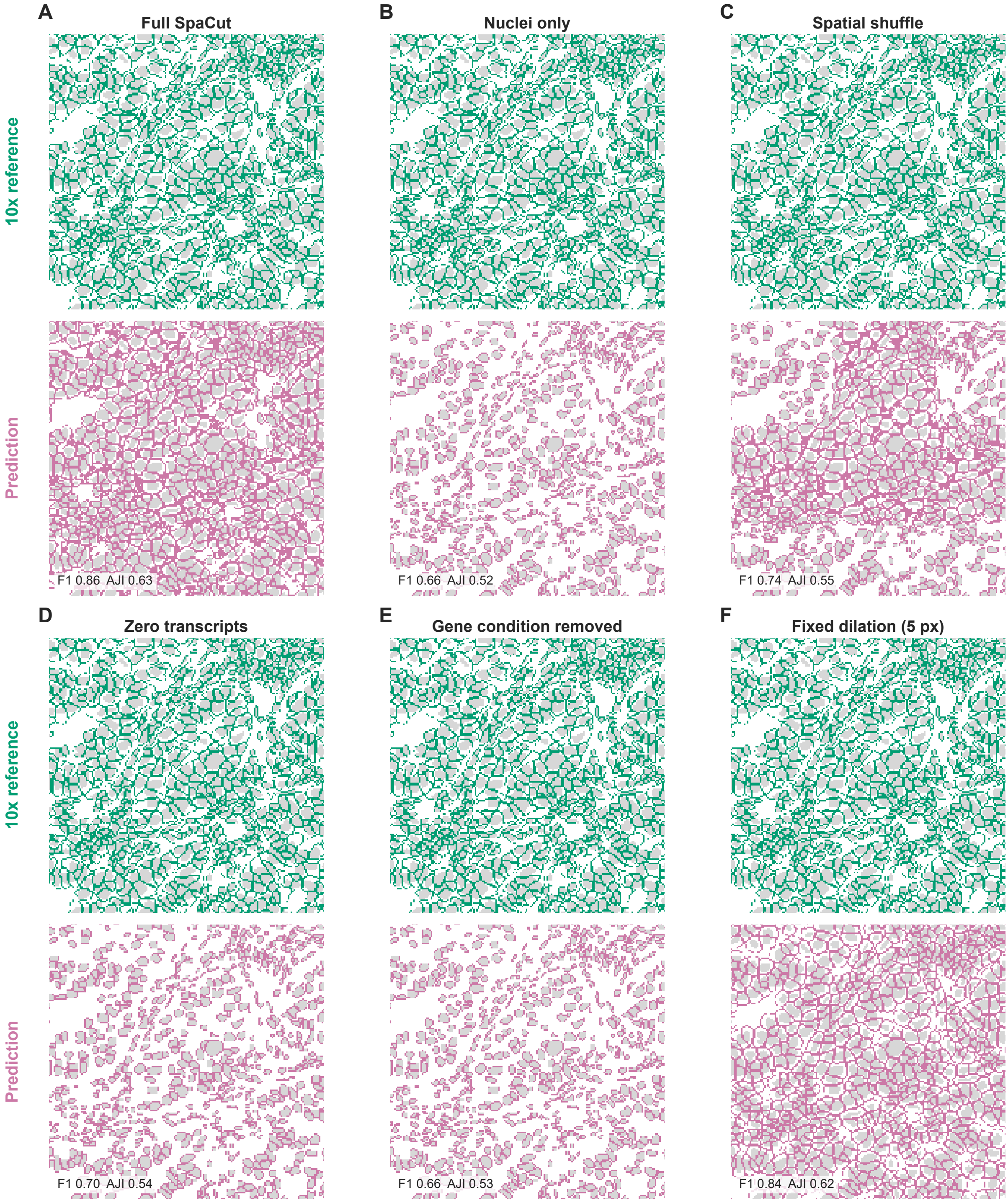

Figure S6: Representative boundaries for the principal controls on Xenium Human Lung Cancer. The same region is shown for all conditions. In each pair, the upper panel shows the 10x reference boundary in green and the lower panel shows the prediction in magenta, with whole-tissue F1 and AJI reported below.

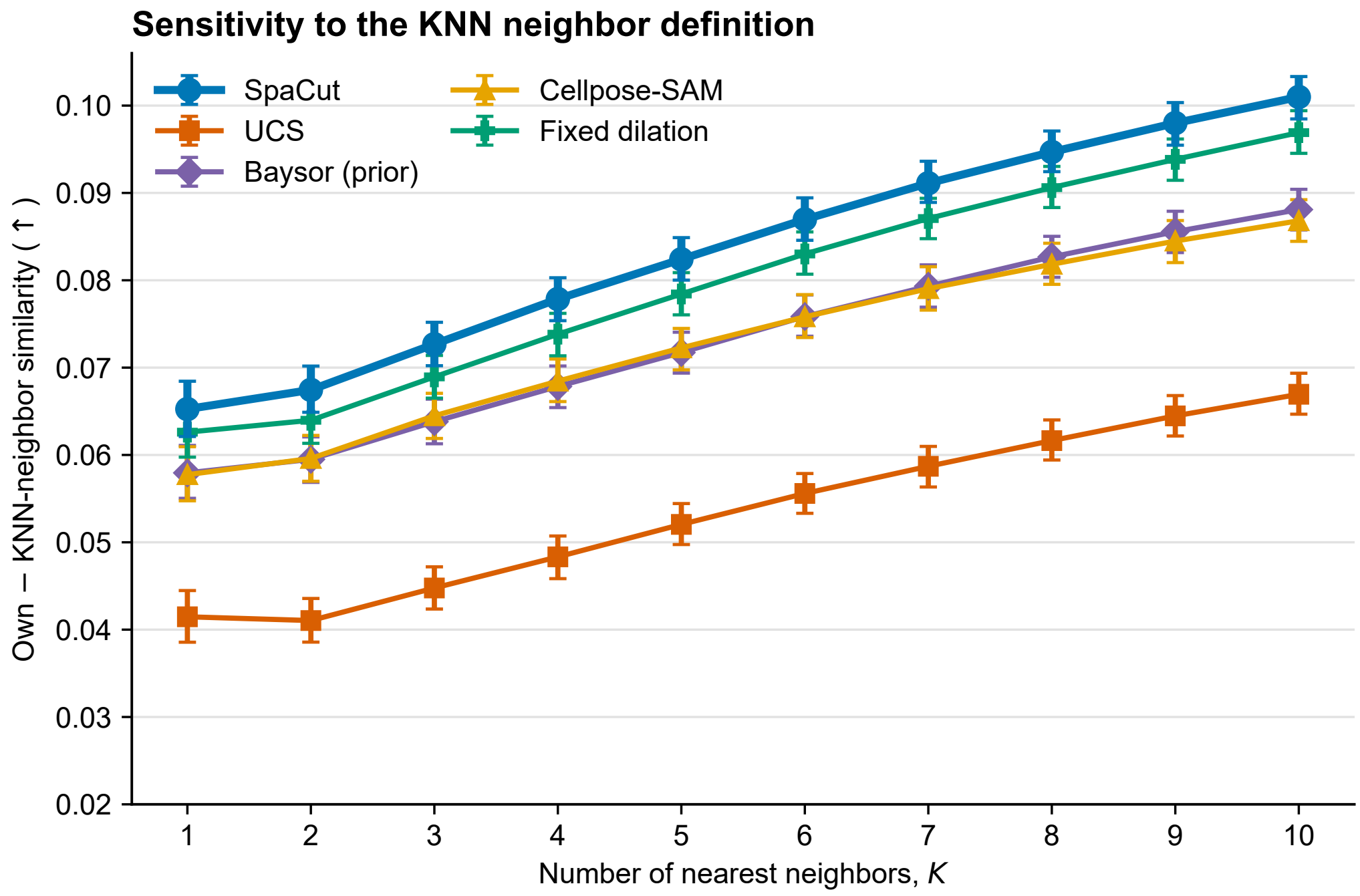

Figure S7: Sensitivity of strict-boundary identity to centroid-KNN neighbor definitions. Cell 10X reference cells are ranked by Euclidean distance between mask centroids. Candidate nuclei with fewer than five transcripts are skipped, and the nearest valid nuclei are included with equal weight for every integer K from 1 through 10. The ordinate is the mean own-nucleus cosine similarity minus the equal-weighted KNN-neighbor cosine similarity; larger positive values indicate stronger own-cell preference. All methods and values of K use the same 12,391 focal cells, and error bars denote 95% focal-cell-bootstrap confidence intervals. SpaCut has the highest mean separation for every tested K. The increase with K across all methods reflects the inclusion of progressively more distant cells and is not interpreted as improved segmentation. This proximity-based analysis is a sensitivity check; the all-direct-contact, contact-length-weighted analysis remains the primary test of boundary-associated signal mixing.

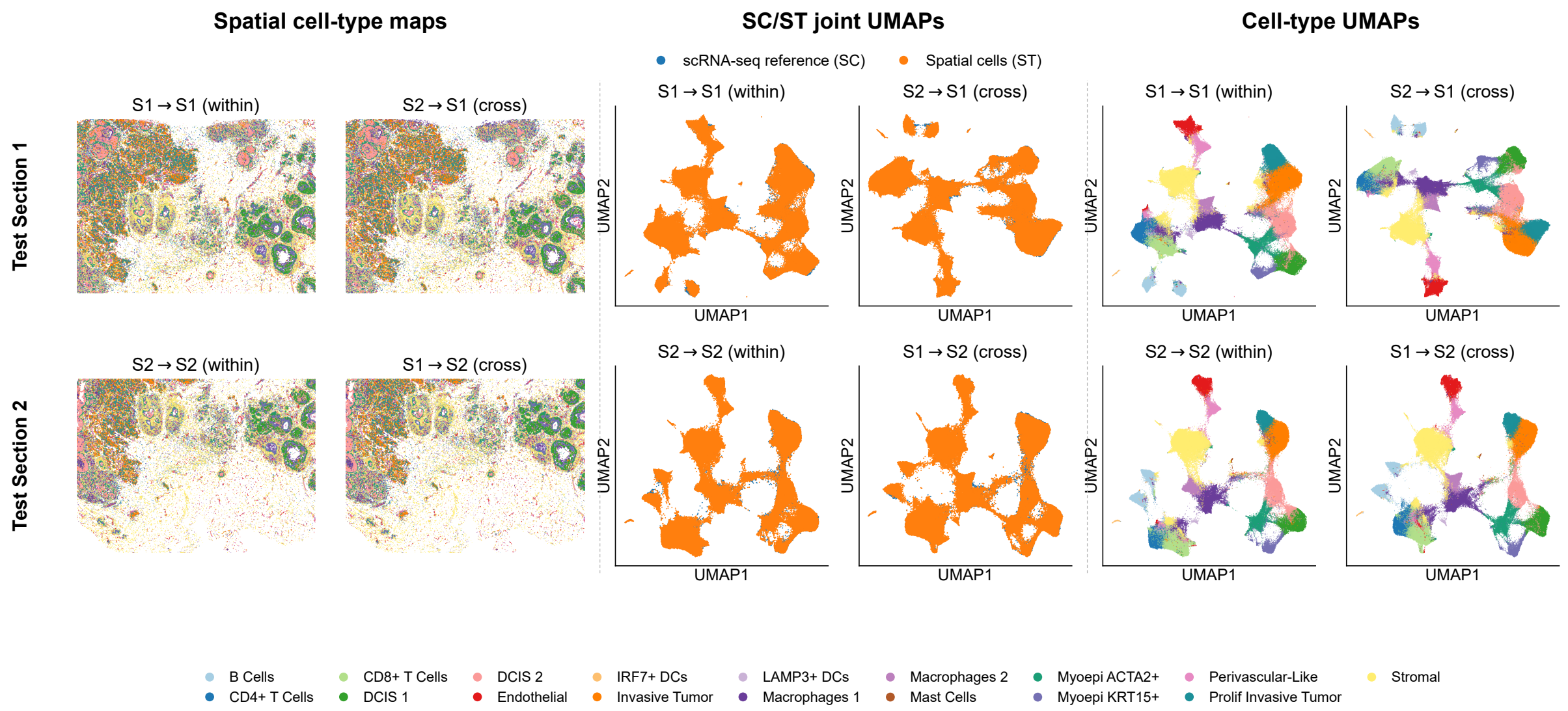

Figure S8: Within- and cross-replicate cell-type annotation on two Xenium Human Breast Cancer sections. For test Section 1, Section 1→Section 1 (within) is compared with Section 2→Section 1 (cross); for test Section 2, Section 2→Section 2 (within) is compared with Section 1→Section 2 (cross). From left to right, paired panels show tissue-wide predicted cell types, joint UMAPs of the scRNA-seq reference (SC) and segmented spatial cells (ST), and the corresponding 17-cell-type annotations. Cross-replicate inference was performed without fine-tuning. All panels retain the original UMAP coordinates and the cell-type palette used throughout the manuscript.

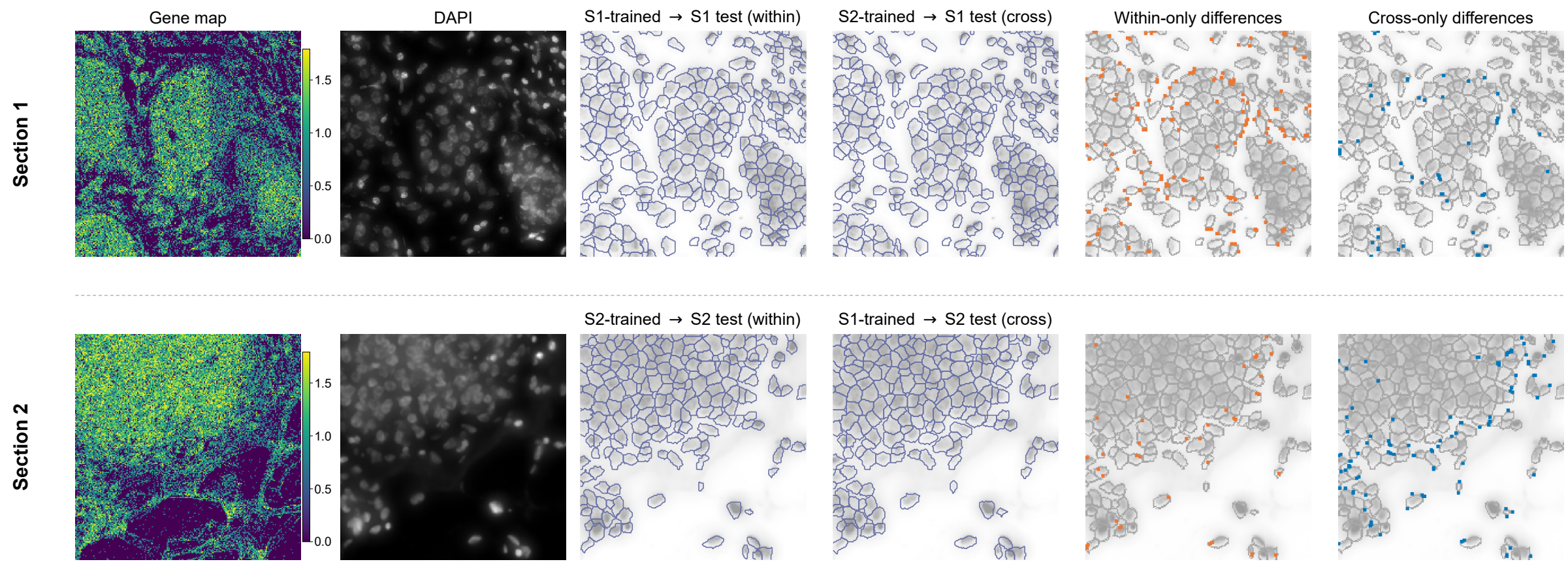

Figure S9: Qualitative cross-replicate consistency of SpaCut on two Xenium Human Breast Cancer sections. The Section 1 row compares Section 1→Section 1 with Section 2→Section 1, whereas the Section 2 row compares Section 2→Section 2 with Section 1→Section 2. Each pair uses the same field of view, gene map, and DAPI image. The last two columns separately display boundary segments found only in the within-replicate prediction (orange) or only in the cross-replicate prediction (blue); matched boundaries within a one-pixel tolerance are shown in light gray as spatial context.

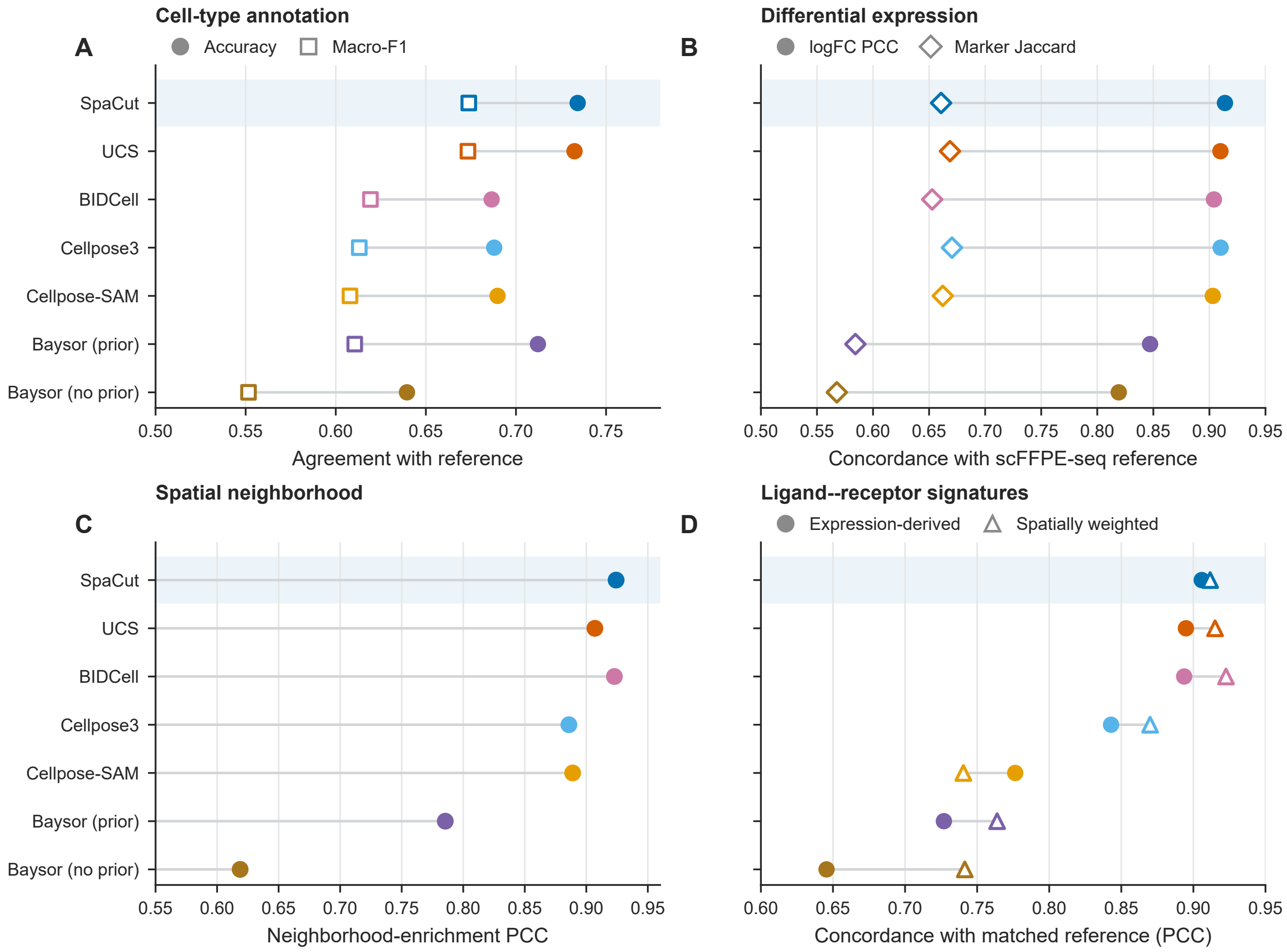

Figure S10: Downstream biological consistency under a common analysis pipeline. (A) Cell-identity preservation, evaluated by annotation accuracy and Macro-F1. (B) Differential-expression concordance, evaluated by mean logFC PCC and marker-gene Jaccard similarity. (C) Agreement of neighborhood-enrichment structure with the supervised spatial reference. (D) Expression-derived and spatially weighted ligand–receptor communication-signature concordance. All panels report the original metric values, and SpaCut is highlighted consistently in blue.

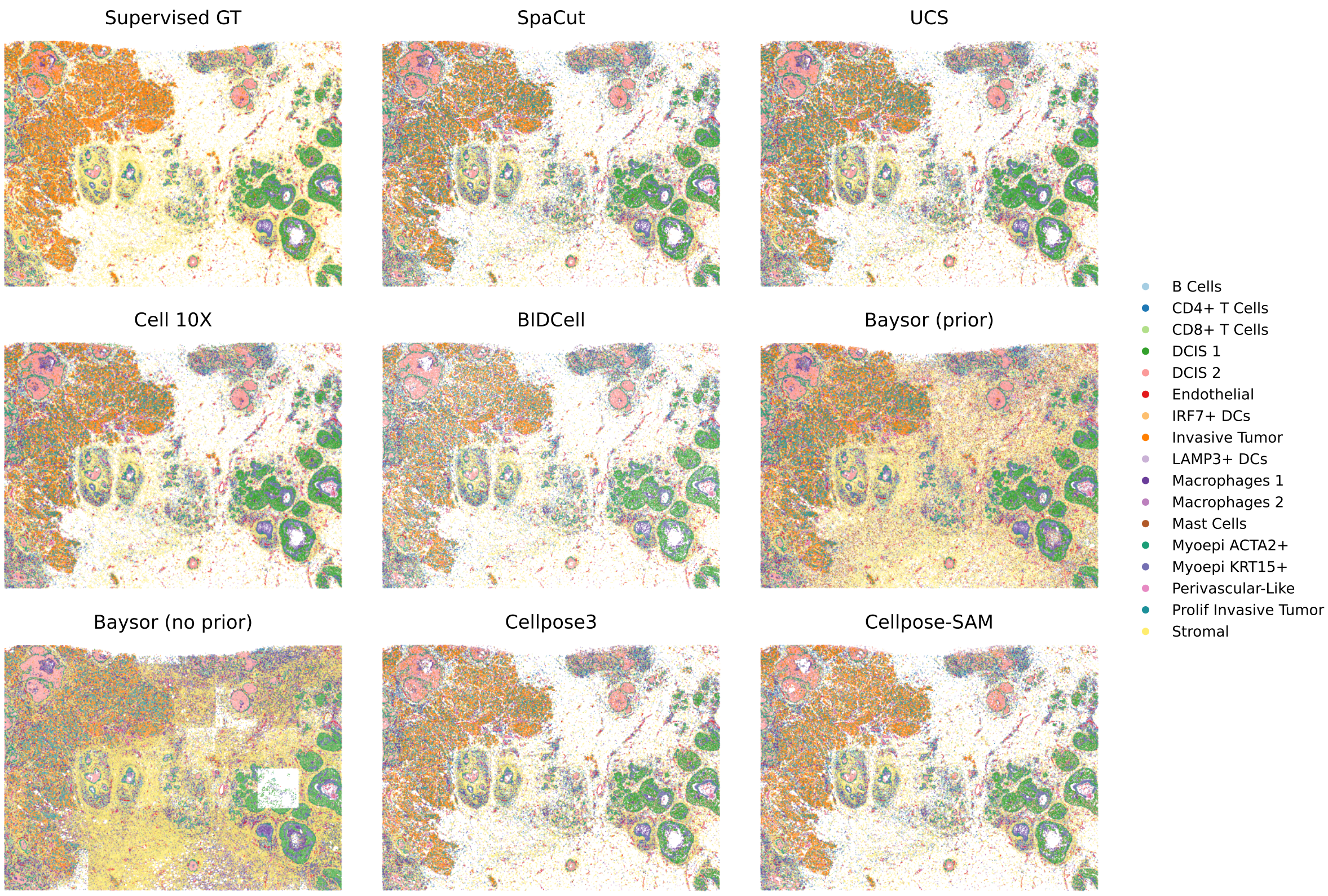

Figure S11: Full-tissue cell-type distributions. Supervised reference labels and predictions from each segmentation-derived cell-by-gene matrix are shown using the same coordinates, cell-type vocabulary, and color mapping. These maps complement the quantitative annotation metrics by revealing whether the predicted cell-type labels retain their tissue-scale spatial organization.

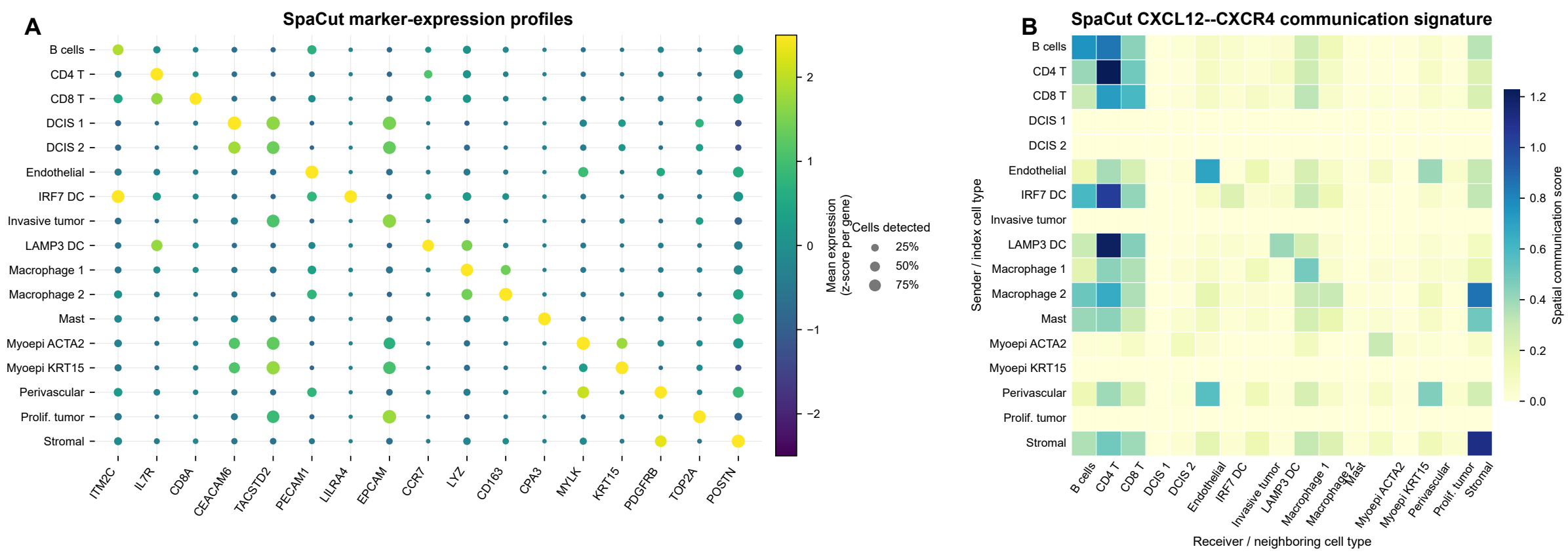

Figure S12: Representative downstream biological readouts obtained from SpaCut segmentation on Xenium Human Breast Cancer. (A) SpaCut marker-expression profiles; one highest-ranked positive and distinct marker is selected per cell type from the completed differential-expression analysis. Dot color denotes mean log-normalized expression standardized within each gene, and dot size denotes the fraction of cells in which the gene is detected. (B) SpaCut-derived spatially weighted sender–receiver communication signature for the representative CXCL12–CXCR4 axis. These panels complement the cross-method quantitative comparisons without repeating the tissue-wide cell-type map or neighborhood-enrichment heatmap.

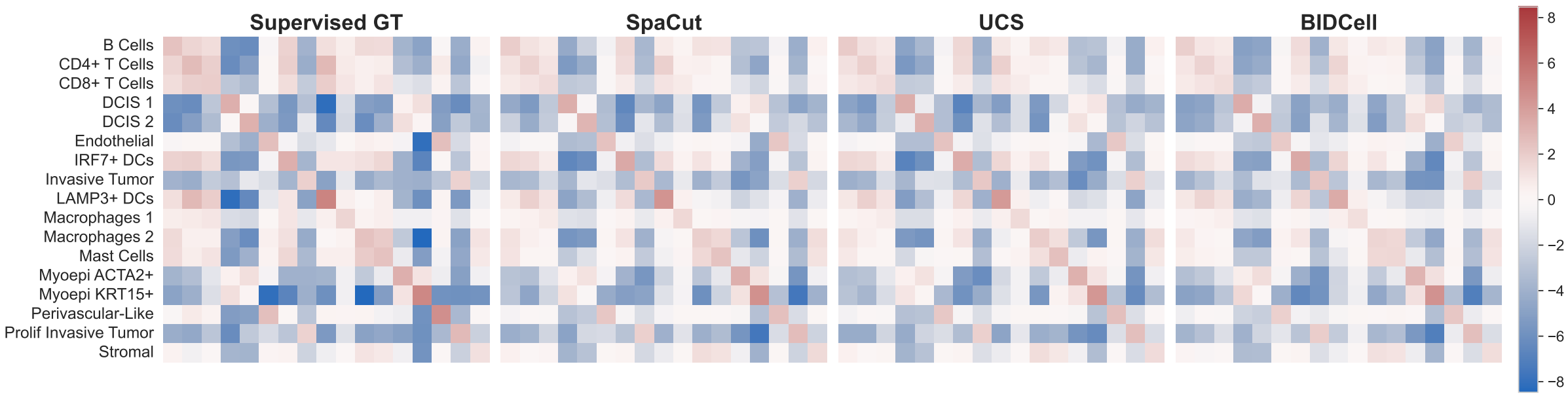

Figure S13: Neighborhood-enrichment structure across segmentation methods. All enrichment matrices are calculated from six-nearest-neighbor graphs with the same 17 cell types. SpaCut most closely reproduces the supervised spatial reference, with the highest PCC (0.9242) and the lowest RMSE (1.1551).

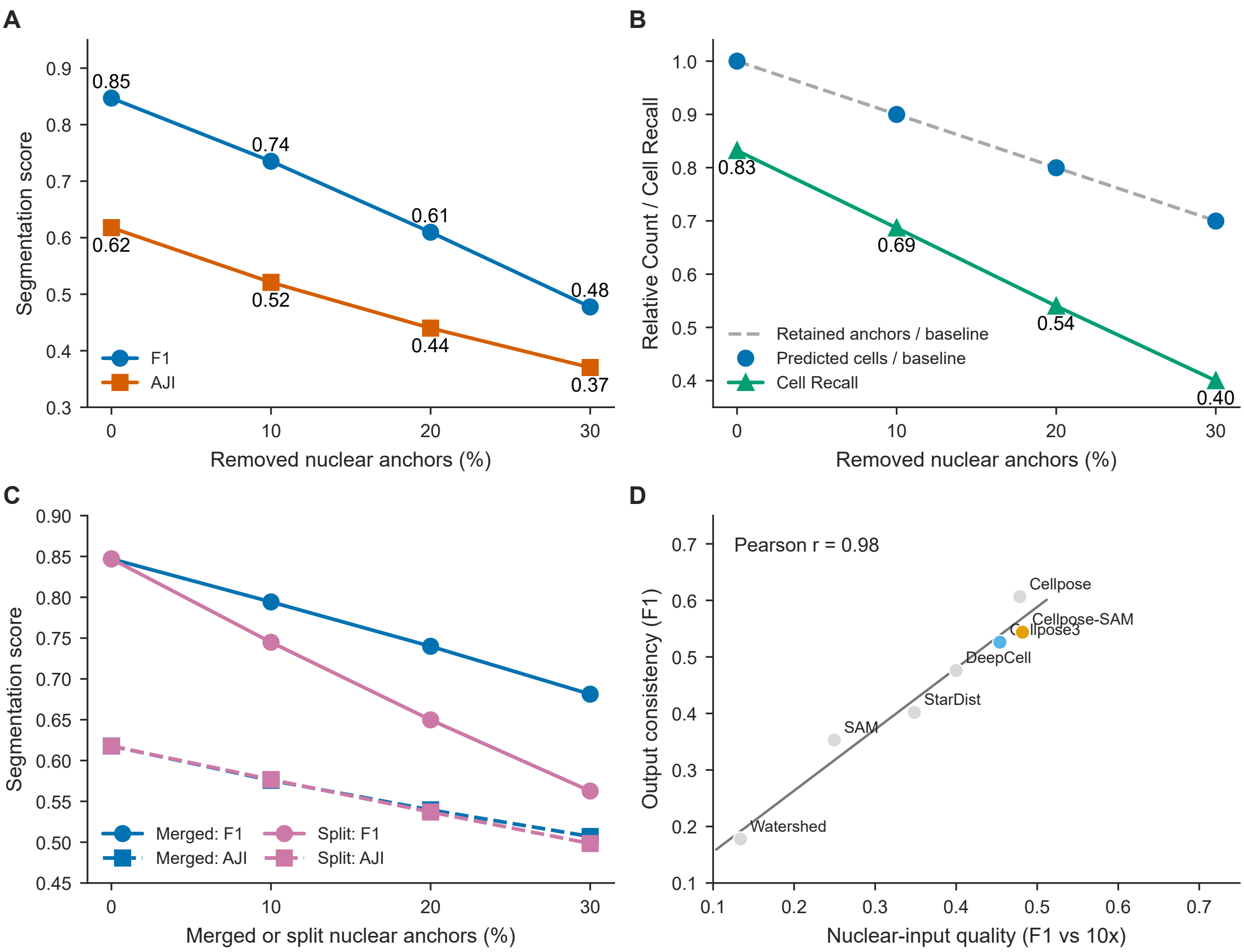

Figure S14: Quantitative sensitivity to nuclear-anchor quality. (A) Whole-tissue instance F1 and standard AJI under nested missed-anchor perturbations. (B) The predicted-cell count relative to baseline closely follows the retained-anchor fraction, while cell recall decreases more rapidly. (C) Dose-dependent effects of merging or splitting 10%, 20%, and 30% of nuclear anchors on instance F1 and AJI. (D) Across seven nucleus segmenters, nuclear-input quality (F1 versus the 10x nuclear reference) is compared with SpaCut-output consistency (F1 versus the official-anchor SpaCut output). Instance F1 uses maximum-cardinality one-to-one matching at an IoU threshold of 0.5.

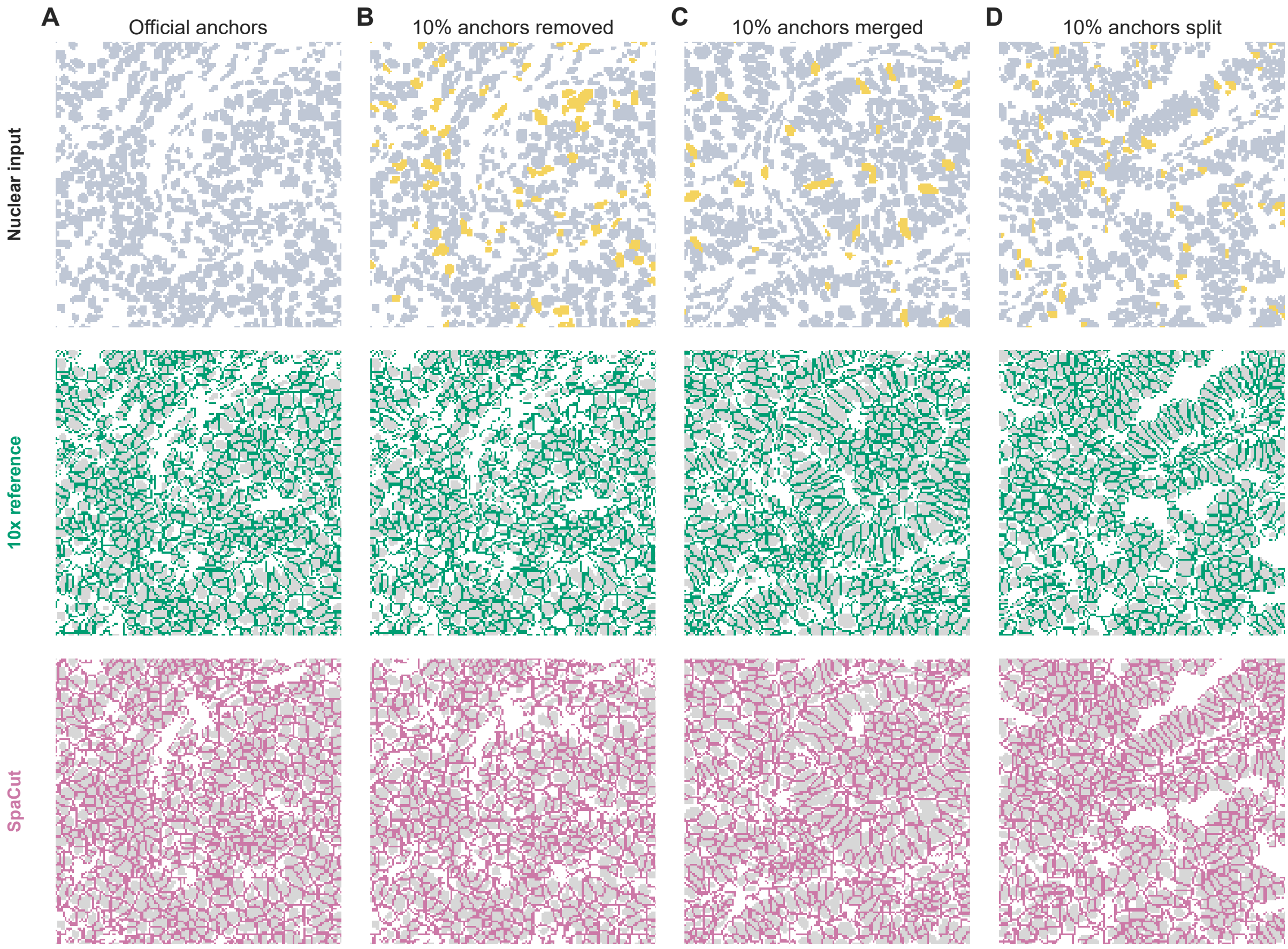

Figure S15: Representative propagation of nuclear-anchor errors. For each condition, the first row shows the official or perturbed nuclear input, with altered pixels highlighted in yellow; the second row shows only the 10x reference boundary (green); and the third row shows only the corresponding full-inference SpaCut boundary (magenta). Reference and predicted contours are deliberately placed in separate panels to preserve local boundary detail. The examples illustrate omission after anchor removal and topological changes after anchor merging or splitting.

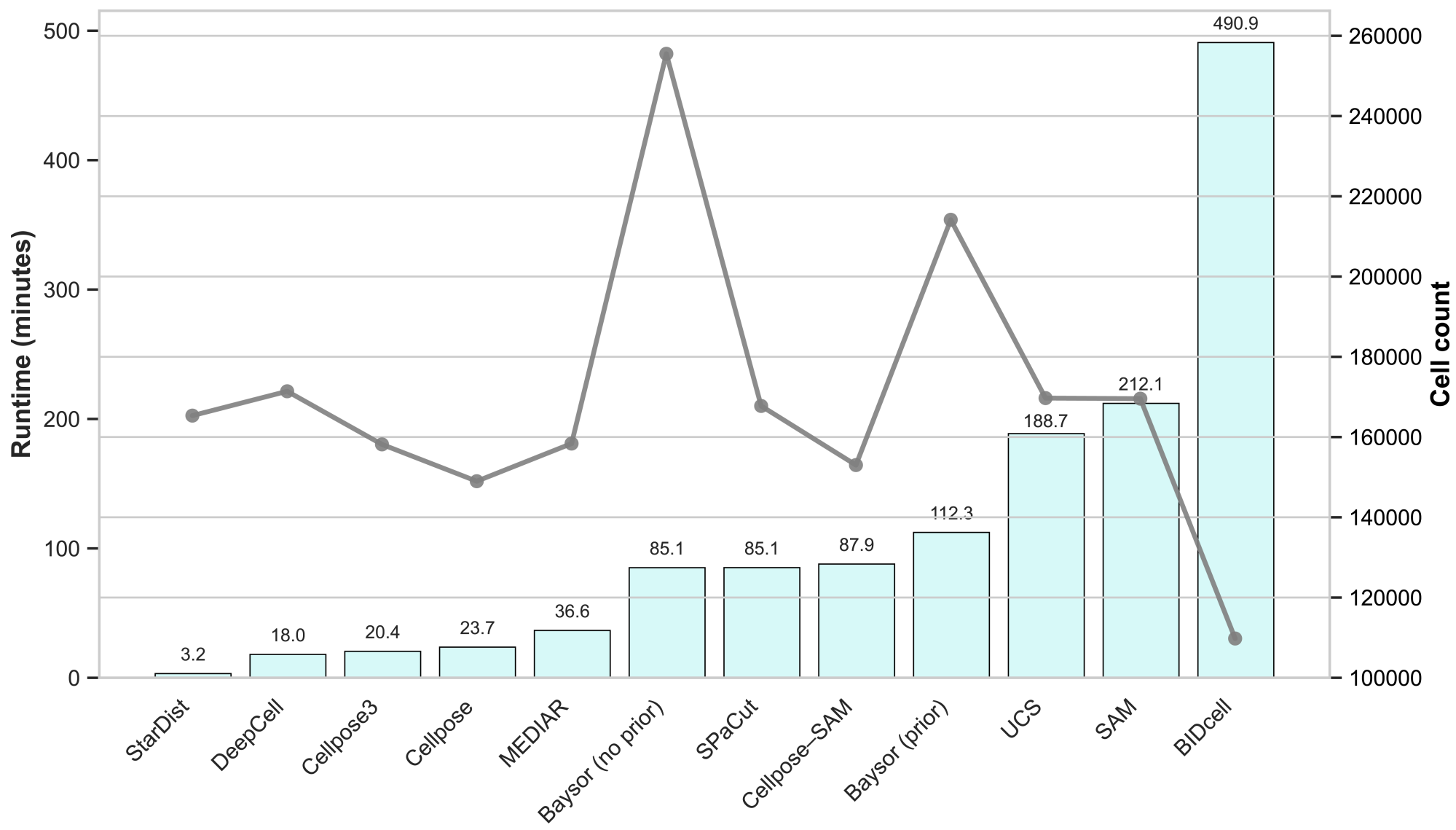

Figure S16: Runtime (bars; left y-axis, minutes) and segmented-cell count (grey line; right y-axis) of representative cell-segmentation methods on the Xenium Human Breast Cancer dataset. SpaCut achieves a favorable runtime–count trade-off, with competitive cell counts at moderate runtime. StarDist is the fastest method in this comparison, whereas BIDCell requires the longest runtime and Baysor (no prior) produces the largest number of segmented instances.

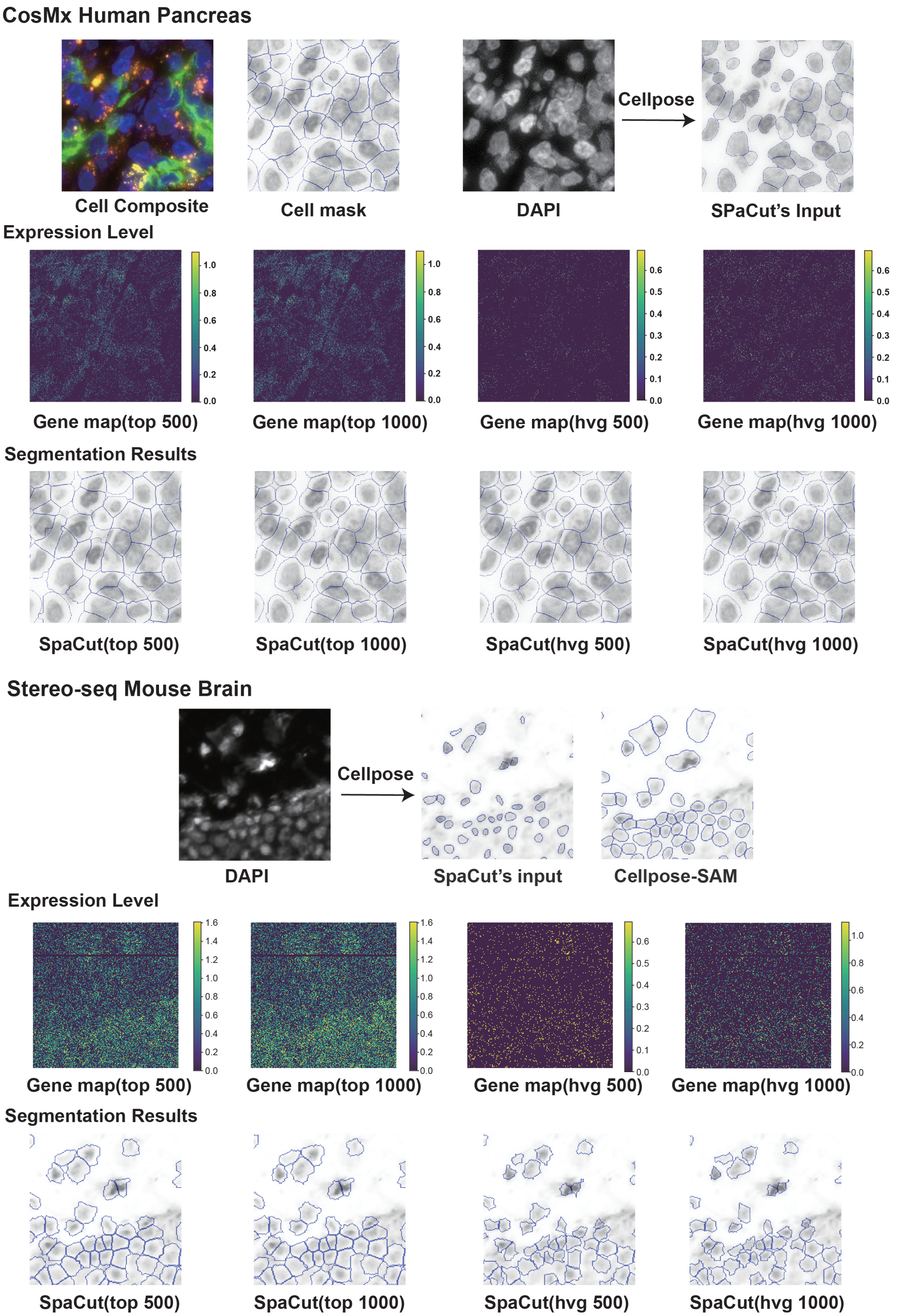

Figure S17: Assessment of gene-set selection for constructing spatial expression maps across two datasets. Top-expressed genes and highly variable genes are compared at set sizes of 500 and 1000 on CosMx Human Pancreas and Stereo-seq Mouse Brain. Maps constructed from top-expressed genes show clearer intensity differences between tissue regions in both datasets. Results are comparable between the top-500 and top-1000 settings; therefore, top-500 is used in subsequent experiments to balance spatial detail and computational cost.

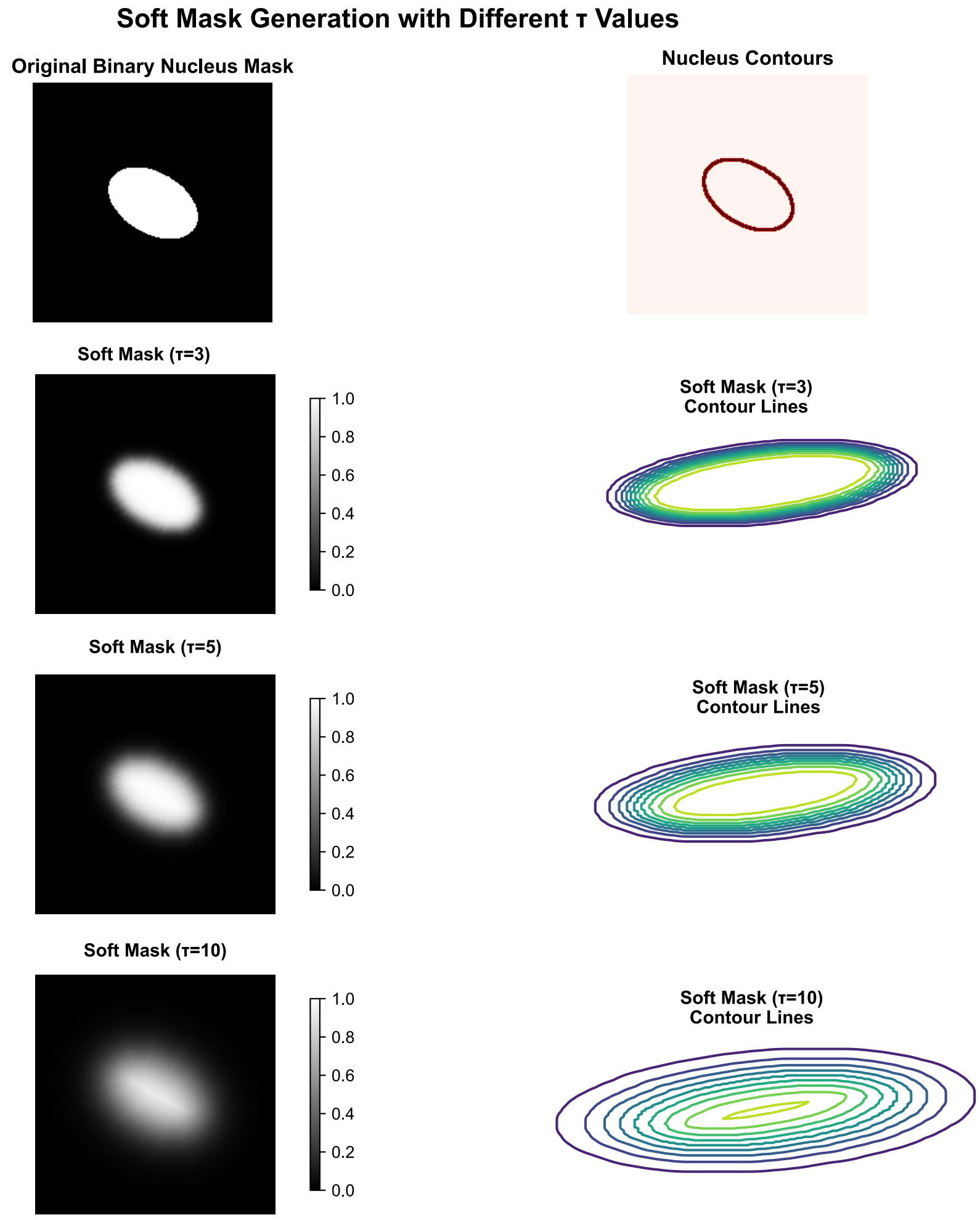

Figure S18: Softmask generation from a binary nucleus mask with different softness parameters τ. The left column shows the original binary nucleus mask and the resulting softmasks for $\tau = 3, 5, 10$, with brighter values indicating higher membership; the right column shows the corresponding isocontours. For a pixel p, $\mathrm{SoftMask}(p) = \sigma(d(p)/\tau)$, where $\sigma(\cdot)$ is the logistic function and $d(p)$ is the signed shortest distance from p to the nucleus contour (positive inside and negative outside). A smaller τ yields a sharper transition, whereas a larger τ produces a smoother and wider boundary halo.
